# Large-Scale Model-Enhanced Vision-Language Navigation: Recent Advances, Practical Applications, and Future Challenges

**DOI:** 10.3390/s26072022

**Published:** 2026-03-24

**Authors:** Zecheng Li, Xiaolin Meng, Xu He, Youdong Zhang, Wenxuan Yin

**Affiliations:** 1 School of Instrument Science and Engineering, Southeast University, Nanjing 210096, China; 220233634@seu.edu.cn (Z.L.); hexu@seu.edu.cn (X.H.); ydzhang@seu.edu.cn (Y.Z.); wenxuan_yin@seu.edu.cn (W.Y.); 2 The China-UK Centre on Intelligent Mobility, Southeast University, Nanjing 210096, China; 3 State Key Laboratory of Comprehensive PNT Network and Equipment Technology, Nanjing 210096, China

**Keywords:** vision-language navigation, large language models, edge deployment, embodied intelligence

## Abstract

The ability to autonomously navigate and explore complex 3D environments in a purposeful manner, while integrating visual perception with natural language interaction in a human-like way, represents a longstanding research objective in Artificial Intelligence (AI) and embodied cognition. Vision-Language Navigation (VLN) has evolved from geometry-driven to semantics-driven and, more recently, knowledge-driven approaches. With the introduction of Large Language Models (LLMs) and Vision-Language Models (VLMs), recent methods have achieved substantial improvements in instruction interpretation, cross-modal alignment, and reasoning-based planning. However, existing surveys primarily focus on traditional VLN settings and offer limited coverage of LLM-based VLN, particularly in relation to Sim2Real transfer and edge-oriented deployment. This paper presents a structured review of LLM-enabled VLN, covering four core components: instruction understanding, environment perception, high-level planning, and low-level control. Edge deployment and implementation requirements, datasets, and evaluation protocols are summarized, along with an analysis of task evolution from path-following to goal-oriented and demand-driven navigation. Key challenges, including reasoning complexity, spatial cognition, real-time efficiency, robustness, and Sim2Real adaptation, are examined. Future research directions, such as knowledge-enhanced navigation, multimodal integration, and world-model-based frameworks, are discussed. Overall, LLM-driven VLN is progressing toward deeper cognitive integration, supporting the development of more explainable, generalizable, and deployable embodied navigation systems.

## 1. Introduction

Developing navigation systems capable of interacting with humans and their surrounding environments remains a long-term objective in Artificial Intelligence (AI) research [1,2]. Instructions that appear simple to humans, such as “walk through the living room and stop at the bedroom door,” still pose significant challenges for autonomous agents. Executing such tasks in complex environments requires human-level competencies, including environmental perception, natural language understanding, interactive communication, planning, and motion control [3].

Visual perception and language interaction constitute essential components of human navigation. As a result, considerable research efforts have been devoted to equipping robots with human-like perceptual and communicative abilities. The underlying motivation is to provide machines with spatial intelligence, enabling them to process spatial and temporal information and to navigate, explore, operate, and make decisions in complex 3D environments such as indoor spaces, low-altitude urban settings, and remote-driving scenarios.

The concept of Vision-Language Navigation (VLN) was introduced by Anderson et al. in 2018 [4]. Unlike traditional map-based navigation approaches, which depend heavily on predefined maps for perception and planning, VLN integrates semantic information from natural language with visual scene representations. This integration facilitates more natural Human–Robot Interaction (HRI) and supports more flexible navigation behavior. The VLN research landscape has since undergone continuous evolution. Early route-following VLN methods relied on explicit routes and landmarks, limiting their applicability in real-world scenarios. Subsequently, goal-oriented VLN emerged, requiring agents to identify targets and explore unknown environments autonomously [5]. More recently, demand-driven VLN has attracted attention, emphasizing semantic reasoning, the interpretation of abstract task descriptions, and decision-making based on commonsense knowledge [6]. This progression reflects a broader shift in VLN from predefined path execution toward semantic understanding, task reasoning, and interactive navigation.

The rapid breakthroughs in Large Language Models (LLMs) and Vision-Language Models (VLMs) are reshaping the VLN landscape [7,8]. Benefiting from their emergent capabilities, these models demonstrate strong contextual modeling, logical reasoning, and cross-modal knowledge integration in Natural Language Processing (NLP) [9,10] and Computer Vision (CV) tasks [11,12]. LLMs, trained on massive-scale corpora, acquire rich linguistic patterns and world knowledge, enabling robust generalization under zero-shot and few-shot conditions [13]. They can leverage commonsense and logical reasoning to make reasonable navigation decisions even without task-specific training. Complementarily, VLMs process multimodal inputs, including images, videos, and 3D environments, to help agents recognize objects, obstacles, and spatial layouts, thereby supporting more efficient path planning and dynamic decision-making. In addition, techniques such as prompt engineering [8,14] and Chain-of-Thought (CoT) reasoning [15,16] enable more interpretable and structured processing of complex tasks. Together, these advantages have positioned LLM-enabled VLN as a central research focus in the community.

Although several surveys have reviewed VLN advances, a systematic analysis of LLM-based VLN remains limited. Most existing reviews focus on conventional VLN techniques or on Real2Sim experiments conducted in controlled environments, with insufficient emphasis on Sim2Real transfer and edge deployment. For instance, ref. [17] provides an overview of LLM applications in robotics but does not analyze VLN in detail. The survey in [18] discusses VLN progress in the era of foundation models, while [19] summarizes traditional path-following and goal-oriented VLN methods. However, these works offer limited coverage of LLM-driven VLN systems. This gap hinders researchers from fully evaluating the practical value of LLM-enabled VLN in real-world scenarios such as indoor/outdoor navigation, low-altitude mobility, and intelligent transportation.

To address these limitations, this paper provides a comprehensive review of LLM-enabled VLN. We analyze how LLMs reshape the VLN framework and summarize State-Of-The-Art (SOTA) advancements in model architectures, algorithms, and task settings. We also review edge deployment literature and real-world applications, highlighting practical constraints and engineering considerations for deploying LLM-based VLN systems. We conclude by outlining key challenges and future research directions.

The remainder of this paper is organized as follows. Section 2 introduces the evolution of VLN technologies. Section 3 reviews SOTA progress in LLM-enabled VLN. Section 4 discusses edge deployment and applications. Section 5 summarizes practical implementation conditions. Section 6 highlights open challenges and future trends. Section 7 concludes the paper.

## 2. Evolution of VLN

From a system perspective, a complete VLN system generally consists of four core components:

- Instruction understanding: Parsing natural-language instructions into structured semantic representations to extract task goals, landmarks, and action sequences. Major challenges include linguistic variability, semantic ambiguity, and long-range dependency modeling;
- Environmental perception: Recognizing objects, spatial layouts, and scene structures from visual inputs and aligning these with language semantics. Challenges include cross-scene generalization, robustness under dynamic conditions, and forming structured representations and long-term memory of the physical environment;
- Planning and decision-making: Generating navigation paths by integrating linguistic intent with perceptual information. Key issues include efficient exploration, avoiding local optima, and improving decision stability;
- Motion control: Executing high-level plans through low-level continuous control. Key challenges include maintaining accuracy, ensuring real-time responsiveness, and mitigating error accumulation during continuous execution.

Built upon these fundamental capabilities, VLN technologies have evolved from geometry-driven approaches to RNN-based architectures and, more recently, to LLM/VLM-driven paradigms. Early geometry-driven methods were, in a strict sense, predecessors of modern VLN. These methods relied primarily on geometric modeling and classical planning algorithms. Simultaneous Localization and Mapping (SLAM), together with graph-based algorithms such as Dijkstra and A*, constituted the core of environmental perception and navigation planning [20,21,22,23]. Motion control was then performed according to task requirements. However, these approaches lacked the ability to understand natural-language semantics, limiting their applicability to HRI tasks.

The formal definition of VLN was introduced by Anderson et al. [4], who described it as enabling an agent to receive natural-language instructions, combine them with visual perception and historical information, and complete navigation tasks in a 3D environment. This requirement marked a shift from geometry-driven to semantics-driven methods, emphasizing the integration of language understanding, visual perception, and motion control. Early VLN systems predominantly adopted sequence-to-sequence frameworks, using RNNs to encode instructions and leveraging reinforcement learning or imitation learning for training. Representative works include Speaker-Follower [24] and Reinforced cross-modal Matching (RCM) [25]. Although these methods achieved strong performance on controlled datasets, they struggled with complex semantic reasoning, cross-environment generalization, and zero-shot learning.

With the rapid advancements in large-scale pretrained models, VLN has entered the LLM-driven stage. In this phase, navigation systems no longer rely solely on task-specific end-to-end models, but instead leverage large models’ abilities in language understanding, cross-modal alignment, and reasoning-based planning. For instance, NavGPT [26] reformulates navigation as a step-by-step language-reasoning process by constructing prompts that include instructions, visual descriptions, trajectory history, and candidate actions. This enables GPT-4 to produce interpretable action decisions and perform complex navigation in zero-shot settings. MapGPT [27] further introduces an “online language map,” constructing and maintaining a dynamic topological representation of the environment in natural-language form. This allows multi-step adaptive planning and enhances global exploration in unknown or partially observable environments. Similarly, NaviLLM [28] proposes a schema-based unified instruction representation, framing navigation, question answering, and trajectory summarization as language-generation tasks. This design enables LLMs to perform cross-task semantic interpretation and decision-making, exhibiting strong task-level generalization across datasets.

Overall, LLM-enabled VLN demonstrates several key advantages:

- Enhanced language understanding and semantic generalization;
- Support for few-shot and zero-shot learning, significantly reducing annotation requirements;
- Cross-task reasoning capabilities, enabling unified handling of navigation, question answering, and object recognition;
- Improved adaptability and decision robustness in open environments.

Despite these advancements, most progress remains constrained to software-level Real2Sim settings [2], with relatively limited research on Sim2Real transfer and real-world deployment. Practical solutions for edge deployment are also scarce [29]. With the growing interest in embodied AI, researchers increasingly recognize that physical embodiments are essential for enabling perception, interaction, navigation, and decision-making in real-world environments [3]. Consequently, moving LLMs from cloud-based settings to edge devices has become an inevitable trend toward achieving embodied intelligence [30]. As autonomous navigation and exploration are core components of embodied systems, LLM-enabled VLN is gradually extending beyond simulation-only development toward integrated hardware–software systems for applications such as low-altitude mobility, indoor/outdoor navigation, and urban transportation.

## 3. Literature Review on LLM-Empowered VLN

**Figure 1:**
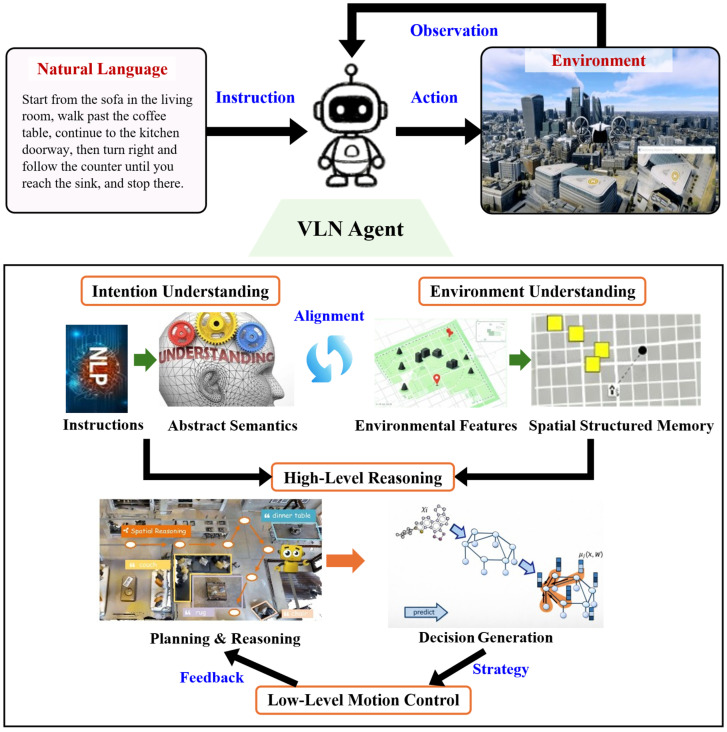
LLM-Enhanced VLN Architecture.

This section systematically reviews the key advances in how LLMs reshape the VLN architecture, specifically divided into four components (as shown in Figure 1): (A) Instruction Understanding, (B) Environment Perception, (C) High-level Planning, and (D) Low-level Motion Planning. These four parts collectively constitute a complete semantic-cognitive-control pipeline from linguistic parsing to physical execution.

Instruction Understanding Module: focuses on natural language semantic parsing and task intent extraction, serving as the linguistic gateway of the entire VLN system.

Environment Perception Module: responsible for visual feature modeling and semantic map construction, providing environmental priors for agent decision-making.

High-level Planning Module: centers on cross-modal reasoning and global path generation, reflecting the agent’s strategic planning and cognitive capabilities.

Low-level Motion Planning Module: realizes the mapping from abstract planning to continuous actions, representing the critical link from perceptual processing to closed-loop control.

The following subsections will delve into the key advances surrounding these four dimensions. To ensure both timeliness and representativeness, this review primarily focuses on studies published within the last five years, which coincide with the rapid emergence and evolution of LLM/VLM-enhanced VLN. Earlier works are included only when they are widely recognized as seminal baselines or paradigm-defining milestones that shaped subsequent VLN research. Across all sections, representative methods are selected to cover key methodological paradigms and application settings, prioritizing works that (i) introduce new problem formulations or architectural frameworks, (ii) report strong performance on commonly used benchmarks and metrics (e.g., SR/SPL/nDTW), (iii) demonstrate notable generalization in zero-shot/few-shot settings, and/or (iv) provide evidence toward practical deployment (e.g., real-world validation, Sim2Real transfer, or edge-oriented considerations).

### 3.1. Instruction Understanding

Instruction understanding is the starting point of a VLN system, with its core objective being to enable the agent to accurately parse natural language instructions and transform them into executable semantic goals or action sequences. This component directly determines whether subsequent perception and planning modules can achieve semantic consistency and behavioral controllability, primarily encompassing two levels: instruction semantic encoding and instruction semantic parsing.

#### 3.1.1. Semantic Encoding

Early works (e.g., Speaker-Follower [24], RCM [25]) primarily adopted the Seq2Seq framework for cross-modal alignment, achieving navigation instruction execution by directly learning mappings from language to action sequences. With the introduction of encoder models like BERT and Transformer, VLN’s instruction understanding capabilities have been significantly enhanced, enabling better modeling of global semantic dependencies. Methods based on such text encoders typically achieve semantic alignment through joint attention mechanisms with visual features, offering concise structures and efficient inference, particularly suitable for resource-constrained or real-time-demanding scenarios.

The Find What You Want (DDN) model [6] employed a demand-driven framework: an LLM first extracts semantic attributes of objects, which are then aligned with CLIP visual embeddings to construct a “demand–attribute–vision” ternary semantic space, thereby retrieving targets in the scene that match natural language requirements. This approach breaks the limitations of traditional category-matching navigation and significantly improves generalization capability and robustness in complex open scenes. LOC-ZSON [31] introduces an object-centric semantic representation approach within the semantic encoding framework. By characterizing complex object concepts via semantic loss functions and combining LLM’s prompt enhancement mechanisms to expand cross-modal alignment capabilities under open-vocabulary conditions, it demonstrates superior performance in both zero-shot retrieval and object navigation (including real-world scenarios).

Ye et al. [32] addressed the challenge of semantic alignment between text and bird’s-eye-view images in cross-view geo-localization, proposing the CrossText2Loc framework. Based on CLIP’s extended functionality, combined with extended positional encoding, OCR and segmentation for hallucination suppression, and an interpretable retrieval modeling pipeline, it effectively enhances the adaptability and robustness of multimodal large models in text-driven geo-localization. Zhang et al. [33], to tackle the challenges of continuous control and open-vocabulary target alignment in UAV VLN, proposed the VLFly framework. Fusing LLaMA-3-8B with CLIP as the core, through collaborative process design of instruction encoding, target retrieval, and waypoint planning, it substantially improves the adaptability and robustness of multimodal large models in monocular RGB continuous velocity output scenarios.

Compared with RNN models commonly employed in traditional early works, Transformer-based text encoders can better capture global semantic dependencies and maintain semantic consistency across multi-turn instructions. To further reduce inference latency and enhance model deployability, researchers have begun exploring pluggable structures for lightweight semantic encoders. For instance, in the DDN system, the text encoding module can operate independently, continuously providing linguistic state updates for the agent, thereby delivering semantic supervision signals with low latency on edge devices. In multi-agent systems, lightweight encoders can also form hierarchical collaboration with LLMs: the former is responsible for rapid semantic capture, while the latter performs high-level reasoning, achieving a balance between efficiency and intelligence. Overall, these methods belong to the instruction understanding paradigm based on semantic encoding, whose core lies in utilizing pre-trained large models to extract contextual semantic embeddings and achieve a unified representation of language and vision through cross-modal alignment, laying the foundation for subsequent semantic parsing.

#### 3.1.2. Semantic Parsing

Unlike traditional encoder-based semantic embedding, LLM-based instruction understanding places greater emphasis on linguistic reasoning and structured expression. As general-purpose language models such as GPT demonstrate powerful logical reasoning and generalization capabilities, an increasing number of studies have begun leveraging LLMs for semantic parsing of natural language and interpretable modeling. Such methods typically enable explicit reasoning and visualization features in the instruction understanding process by generating intermediate semantic representations or introducing chain-of-thought mechanisms.

InstructNav [34] introduces the Dynamic Chain of Navigation (DCoN) mechanism that integrates different task types through a unified linguistic planning process. It also combines multi-sourced value maps to map linguistic plans into executable trajectories in real time, achieving stronger zero-shot generalization and task transfer capabilities. Long et al. [35] proposed the Discuss Before Moving (DBM) framework, which performs semantic reasoning and consensus aggregation before execution through a multi-expert discussion mechanism, thereby enhancing the accuracy and interpretability of instruction understanding in VLN.

NaviLLM [28] adopts a schema-based instruction strategy, unifying navigation, target localization, history summarization, and visual question answering into a generative modeling framework, achieving semantic sharing and unified training across multiple tasks. GC-VLN [36] parses natural language instructions into graph-structured constraints containing entities and spatial relationships, enabling training-free semantic–spatial mapping. This method constructs structured semantic representations in the form of “instruction→graph constraint→path solving”, making language understanding executable and interpretable, and providing explicit semantic constraints for subsequent planning modules. Wang et al. [37] propose an instruction-aware planning framework in which semantic cues extracted from the instruction are aligned with candidate paths on a 3D semantic map. This implicit form of semantic parsing allows the system to incorporate language-derived hints into the path-scoring process, improving instruction compliance without relying on explicit LLM-based structured representations.

These methods collectively drive a paradigm shift in VLN from language matching to semantic reasoning: models no longer merely identify keywords but establish structured semantic mappings of “instruction–environment–action”. In this process, LLMs act as a “cognitive interpreter”, enabling natural language parsing to possess reasoning capability, transferability, and interpretability, marking a new stage where instruction understanding evolves from static semantic encoding to dynamic semantic reasoning.

Category comparison and application scenarios. Semantic encoding uses pretrained text encoders and cross-modal alignment to produce compact representations, making it efficient and deployment-friendly for real-time or resource-constrained systems, but it is often limited in explicit reasoning for complex compositional constraints. Semantic parsing leverages LLMs to generate interpretable intermediate representations (e.g., structured subgoals), improving instruction faithfulness and generalization, yet it typically incurs higher latency and can be sensitive to prompting. Overall, semantic encoding is preferable when efficiency is critical, whereas semantic parsing is more suitable when interpretability and complex instruction understanding are required. Table 1 summarizes representative methods for instruction understanding.

### 3.2. Environment Understanding

Beyond instruction parsing and understanding, agents must construct structured feature descriptions of the external environment to support high-level planning and low-level control. Environment perception involves not only the comprehension of static semantics but also dynamic structured memory and generative prediction. Its core objective is to transform raw visual inputs into semantically consistent spatial representations, enabling agents to form stable and interpretable world cognition in complex scenes.

With the rapid development of VLMs, spatial projection mechanisms, and generative modeling, environmental modeling has evolved from 2D perception to 3D semantic mapping and from passive recognition to active prediction. Overall, research on environment perception can be divided into three levels: (1) environmental feature extraction; (2) structured memory; (3) active generative prediction.

#### 3.2.1. Feature Extraction

Agents need to extract key features from multi-view, complex visual inputs and transform them into semantic representations consistent with language instructions. VLM-based methods fully leverage the semantic abstraction and cross-modal alignment capabilities of large-scale pre-trained models, achieving semantic-enhanced perception through language–vision collaboration. LangNav [38] combines pre-trained VLM with detector modules as a semantic enhancement scheme, enabling the model to form language-consistent spatial understanding in complex scenes. LLM-guided exploration proposed by Dorbala et al. [39] improves reasoning efficiency while maintaining recognition accuracy. ESC [40] achieves zero-shot exploration through soft commonsense constraints. MapGPT [27] presents an online semantic map generation mechanism that transforms visual inputs into language prompts fed into LLM, thereby achieving synergy between perception and reasoning. The method proposed by Qiu et al. [41] unifies spatial and semantic representations with open-vocabulary 3D semantic maps and introduces dynamic replanning mechanisms, enabling the system to complete language-driven mobile manipulation in unseen environments. LLVM-Drone [42] combines LLMs with vision models to refine UAV scene understanding through structured prompts and consistency checks, extracting more reliable semantic environmental features without building long-term maps. LM-Nav [43] utilizes CLIP [44] to align landmark descriptions with visual observations, achieving more accurate target matching and semantic navigation. Wu et al. [45] introduced the DuAl-VLN task and the AeroDuo framework, a Qwen2-VL-based dual-UAV collaborative system. By integrating high-altitude semantic mapping with low-altitude lightweight obstacle avoidance and relying on minimal coordinate exchange, it markedly enhances the adaptability and robustness of multimodal large models in complex urban environments. These studies drive the transformation of perception modules from passive recognition to semantic interpretation and spatial reasoning, enabling agents to comprehend environmental states within a unified semantic space.

However, while pure VLM models possess strong semantic expression capabilities, they suffer from high inference costs and limited real-time performance. Consequently, some research has shifted toward efficient feature modeling methods based on visual encoders. NaviD [46] performs temporal encoding of historical and current image sequences via EVA-CLIP to achieve a continuous visual representation. NavGPT-2 [47] employs lightweight visual projectors or adapters (e.g., Q-Former/Adapter) to map visual inputs into fixed-length tokens for joint reasoning with LLM. Liu et al. [48], focusing on the challenge of fine-grained landmark matching in panoramic images for urban aerial VLN, proposed the NavAgent framework. Based on GLIP, it designs a core workflow through collaborative landmark text extraction, cross-view visual recognition, and topological graph global encoding, significantly enhancing the adaptability and robustness of multimodal large models (MLLMs) in UAV street-view navigation. For the challenge of long-sequence visual–semantic coupling in city-scale VLN, the FLAME [49], based on Flamingo, designs optimization processes, including single-view description learning, multi-view integration and route summarization, and end-to-end action prediction, substantially enhancing the adaptability and robustness of MLLMs in outdoor navigation scenarios. Zeng et al. [50], targeting the challenge of extremely small imaging for distant targets in zero-shot outdoor object navigation, proposed the EZREAL framework. Relying on a multi-scale image pyramid to build its core architecture, through technical combinations of hierarchical saliency fusion, visibility-aware memory, and depth-free heading estimation, it significantly enhances the adaptability and robustness of multimodal large models in long-distance navigation.

Additionally, Li et al. [51] introduced predictive reconstruction mechanisms into visual encoders, enabling models to extrapolate future states from historical observations and possess prospective understanding capabilities. This trend indicates that environment perception is transitioning from passive recognition to dynamic prediction, laying a semantic foundation for subsequent structured modeling and generative reasoning.

#### 3.2.2. Structured Memory

To enhance agent consistency and environmental understanding capabilities over long time horizons, researchers have integrated memory mechanisms with structured semantic representations, evolving environmental representations from transient perception into continuously updateable spatio-temporal models.

OVER-NAV [52] constructs a compact Omnigraph that integrates open-vocabulary detections with LLM-parsed semantics, serving as a persistent cross-round memory. This structured representation preserves key entities and relations, improving long-horizon consistency in Iterative VLN (IVLN). Zhang et al. [53], to address the combinatorial explosion in planning caused by long trajectories and large action spaces in urban aerial VLN, designed the CityNavAgent framework, building its core upon GPT-4V and combining collaborative mechanisms of open-vocabulary perception, hierarchical semantic planning, and global memory maps to effectively enhance the adaptability and robustness of multimodal large models in continuous 3D aerial navigation. Zeng et al. [54] proposed JanusVLN, which reduces redundancy and enhances generalization through semantic/spatial dual implicit memory. Zhang et al. [55] proposed COSMO, introducing a selective memory mechanism that reduces computational cost through cross-modal state sparsification. Song et al. [56] proposed Guide-LLM, which explicitly models environmental topology with textual nodes and edges, enabling LLMs to access and update navigation semantics in linguistic space. Wang et al. [57] proposed Dynam3D, which introduces dynamic hierarchical 3D tokens for semantic–geometric coupling, online updating, and long-term 3D environment memory across navigation tasks.

Furthermore, recent work has introduced temporal consistency and knowledge-augmented modeling: VLN-KHVR [58] integrates external knowledge with navigation history to construct temporally consistent representations. VLN-ChEnv [59], for dynamic and changeable environments, proposes multimodal temporal modeling and semantic updating mechanisms. StreamVLN [60] adopts a SlowFast streaming architecture, where the fast pathway captures real-time changes, while the slow pathway maintains long-term consistency.

These methods collectively drive the evolution of structured memory from static topology to dynamic temporal modeling, enabling agents to maintain semantic stability and task robustness during long-term interactions.

#### 3.2.3. Active Generative Prediction

For agents, the ultimate goal of environment perception is not merely to understand the observed world, but to actively generate and predict the unobserved world. Generative semantic construction emerges as a new direction, endowing agents with the capability to “imagine–simulate–correct” and enabling forward-looking decision-making.

VLFM [61] introduces linguistic frontier regions in semantic maps to achieve active exploration and zero-shot navigation based on semantic prediction. Huang et al. [62] proposed VISTA, adopting an imagine-and-align strategy: under linguistic and current visual conditions, diffusion models generate visual imagination, which is then matched with real observations through an alignment module, enhancing navigation intelligence in partially observable scenes. Fan et al. [63] fused scene graphs and voxel features to optimize prompts, making generated instructions more aligned with task contexts. ImagineNav [64] achieves integration from understanding to reconstruction through generative scene reconstruction and semantic alignment. Saanum et al. [65] used simplified world models to predict future states, validating the feasibility and interpretability of world models in complex decision-making tasks. Huang et al. [66], addressing the difficulty of coupling global and local aspects in long-range language navigation in open environments, proposed the KiteRunner framework. Centered on MLLM, it builds a collaborative architecture that substantially enhances MLLM’s adaptability and robustness in outdoor complex scenes through a synergistic mechanism of linguistic semantic parsing, UAV orthographic image modeling, and diffusion model-based local trajectory generation.

Generative modeling not only fuses linguistic priors with visual observations but also actively generates predictive representations in latent space, forming a “prediction function” for the perception system. This marks the transition of perception systems from passive observation to cognitive simulation, laying the foundation for closed-loop embodied intelligence.

Category comparison and application scenarios. Feature extraction uses VLMs or efficient visual encoders for language-aligned semantic cues, benefiting open-vocabulary perception and cross-scene generalization, but incurring high computational costs for continuous long-horizon processing. Structured memory maintains persistent semantic–spatial representations (e.g., graphs and maps), improving long-term consistency and global exploration, though with added memory management complexity. Active generative prediction equips agents with anticipatory capabilities by “imagining” unobserved states, which is promising under partial observability, yet requiring heavier models and careful control of model/prediction mismatch and hallucination. Table 2 summarizes representative methods for environment understanding.

### 3.3. High-Level Planning

High-level planning serves as the core component in VLN that bridges semantic understanding and action control. Its objective is to generate globally executable planning strategies based on instruction semantics and environmental perception results. Unlike traditional rule-based or reinforcement learning path-search methods, LLM-based planning transcends direct “state-to-action” mapping, instead possessing capabilities such as explicit reasoning, semantic interpretation, and generative planning. This enables agents to form decision-making chains characterized by logical reasoning across diverse tasks and complex scenarios.

From an evolutionary research perspective, high-level planning has undergone three main stages: (1) planning based on explicit logic and interpretable strategies; (2) planning based on implicit representations and adaptive strategies; and (3) generative planning based on world models. These methods collectively drive the transformation of VLN technology from “experience-driven decision-making” to “cognition-driven reasoning,” gradually endowing agents with human-like semantic understanding and reasoning capabilities that better align with the requirements of embodied intelligence.

#### 3.3.1. Explicit Reasoning

Explicit reasoning represents one of the primary forms of LLM involvement in VLN planning. Its core idea is to achieve interpretable decision path construction through language generation or structured intermediate representations. Unlike traditional reinforcement learning that relies on implicit policy networks, explicit reasoning provides transparent task decomposition and strategy interpretation at the reasoning chain level, making each step of the model’s planning traceable and reviewable.

EvolveNav [67] enhances explicit reasoning by enabling LLMs to iteratively refine their own reasoning chains through a self-improving loop, leading to more reliable and coherent embodied decision-making. MSNav [68] integrates LLM-based spatial reasoning with a lightweight dynamic memory. The system queries the LLM to explicitly infer object relations and directional cues from the instruction, and aligns these semantics with accumulated scene observations. This explicit reasoning process provides clearer guidance for decision-making and supports zero-shot navigation in unseen environments. NavGPT [26] adopts a chain-of-thought prompting mechanism, decomposing complex navigation goals into explicit sub-intent sequences and generating step-by-step action descriptions at the textual level, achieving stronger semantic interpretability. NavCoT [69] incorporates a Navigational CoT mechanism, enabling the LLM to first imagine the next observation, then filter matching frames, and finally generate actions at each navigation moment, thereby enhancing the reasoning depth and interpretability of path planning. PaLM-SayCan [70] adopts a “language agent–execution agent” collaborative architecture, where the LLM is responsible for high-level strategic planning, while the underlying robot module executes actions and feedback verification. This explicit task decomposition and closed-loop mechanism makes the planning process more akin to human decision-making logic, first generating linguistic plans, and then continuously refining strategies based on environmental feedback, thus improving agent interpretability and execution reliability in complex tasks. Khan et al. [71], to address the challenge of multi-source uncertainty adaptation for UAVs in dynamic environments, proposed a DeepSeek-v3-based context-aware navigation algorithm, designing a decision-making process supported by multimodal sensor data. Through technical combinations of weighted goal fusion, interpretable direction scoring, and sixteen-direction discrete decision-making, it significantly enhances the adaptability and robustness of multimodal large models in complex scenarios.

Furthermore, VLN-Zero [72] extends explicit reasoning to a neuro-symbolic fusion paradigm. This method rapidly constructs semantic scene graphs through structured language prompts and generates executable paths using a cache-enhanced neuro-symbolic planner, achieving efficient transfer and interpretable decision-making in zero-shot scenarios. Its core innovation lies in integrating language parsing, semantic modeling, and planning solving into a unified framework, pushing explicit reasoning toward generative world modeling. Meanwhile, FSR-VLN [73] builds explicit semantic structures based on hierarchical multimodal scene graphs, introducing a fast–slow cascaded reasoning mechanism to balance global efficiency and local precision, further strengthening hierarchical semantic expression in the planning process and demonstrating the trend of integrating explicit reasoning with generative modeling. Qiao et al. [74], addressing the issues of high cost and weak spatial reasoning in closed-source models for zero-shot continuous VLN, constructed the Open-Nav framework, extending functionality based on Llama3.1-70B. Through synergistic mechanisms of waypoint prediction, scene perception, and spatiotemporal chain-of-thought reasoning, it effectively enhances the adaptability and robustness of open-source large models in real-world indoor/outdoor continuous navigation.

However, explicit reasoning models often face high computational overhead and inference latency, limiting their real-time performance and deployability. To this end, research has gradually shifted toward implicit planning mechanisms that implement semantic-action mapping in latent space.

#### 3.3.2. Implicit Reasoning

In contrast to explicit reasoning, implicit reasoning emphasizes adaptive decision-making for complex tasks through continuous state evolution and policy generation in latent space. Such methods typically do not explicitly generate intermediate linguistic descriptions, but instead establish an intrinsic mapping of “semantics–perception–action” in latent space through multimodal joint learning, thereby enabling efficient inference in low-latency scenarios.

NavGPT-2 [47] adopts lightweight visual projectors such as Q-Former or Adapter (referencing InstructBLIP’s architecture) at the LLM input stage, compressing multi-view visual features into fixed-length tokens before feeding them into the language model for cross-modal reasoning, achieving direct decision generation without explicit textual reasoning. The input-adaptive inference framework proposed by Kang et al. [75] achieves efficient path reasoning in latent semantic space through a confidence-driven dynamic computation mechanism, significantly reducing inference overhead and latency in VLN models. Liu et al. [76] proposed the Energy-Based Policy (EBP) framework, which models VLN as an energy minimization problem. By measuring the consistency among states, actions, and instructions through a cross-modal energy function, it achieves implicit policy generation under semantic constraints in latent space, effectively enhancing policy stability and cross-scene generalization capability. Qi et al. [77] optimized VLM into an end-to-end continuous navigation policy through reinforcement fine-tuning, balancing global objectives and immediate feedback using a time-decay reward function, thereby achieving adaptive policy evolution in latent space.

Additionally, Pixel-Guided Navigation Skill (PGNS) [78] belongs to the category of implicitly semantic-guided policy learning methods. This approach learns the correlation distribution between language and pixel features in visual semantic space to guide action policy generation, achieving end-to-end generalization from perception to control in zero-shot object navigation. PGNS does not rely on explicit reasoning chains but implicitly influences decision-making through semantic features, representing a transitional direction from linguistic reasoning to perception-driven policy learning. Liu et al. [79], addressing the issues of excessively long 4-DoF continuous-space paths and action space explosion in aerial VLN, constructed the AerialVLN benchmark and proposed the LAG training strategy. Based on CMA and adopting a progressive fine-tuning approach, it effectively enhances the adaptability and robustness of cross-modal models in urban aerial navigation through a combination of human flight sampling, lookahead guidance, and modality ablation.

Overall, implicit reasoning improves efficiency and often offers strong generalizability in latent space, but its reduced interpretability makes error attribution and safety auditing more challenging. Recent work, therefore, explores visualization and externalization techniques to make implicit structures more transparent.

Although explicit and implicit reasoning paradigms are clearly distinguishable, in practice, they often need to trade off against runtime performance and deployability. Explicit reasoning enables interpretable decisions valuable for debugging and safety, but increases latency and cost. Implicit reasoning achieves higher throughput and lower latency, yet sacrifices explainability. Crucially, higher interpretability does not guarantee better overall performance, especially under real-world constraints like edge compute, energy budgets, and safety requirements. Hybrid planning offers a promising solution, using explicit reasoning for high-level goal decomposition and error diagnosis, while lightweight implicit policies handle frequent low-level decisions. Alternatively, selective reasoning defaults to efficient implicit inference, invoking explicit methods only during uncertainty (e.g., low confidence or repeated failures). Such designs balance interpretability, robustness, and efficiency, improving both benchmark performance and deployment readiness.

Beyond this interpretability–efficiency trade-off, world-model-based generative planning further introduces anticipatory decision-making by simulating future trajectories in latent space, which is discussed next.

#### 3.3.3. Generative Planning

With the deep integration of embodied intelligence and generative modeling, researchers have begun exploring paradigms that combine high-level planning with world models. World models enable agents to perform imaginative decision-making in latent space by learning environmental state transitions and reward functions, allowing them to complete multi-step reasoning and policy generation without direct interaction with the real environment. This concept signifies the evolution of planning systems from reactive decision-making to generative reasoning.

Cog-GA [80], centered on LLMs, constructs a “generate–execute–reflect” closed loop in continuous environments. This model maintains task context through semantic memory and self-corrects during execution, achieving language-driven generative reasoning and interpretable planning, marking VLN planning’s advancement toward cognitive-featured generative decision-making. Ha et al. [81] proposed the world models framework to learn the temporal evolution of environments through latent dynamic models, enabling agents to simulate future states internally and thus realize imagination-based planning and control. In VLN scenarios, the Dreamwalker [82] introduces a world model structure that embeds language-guided semantic planning into latent dynamic prediction modules, implementing a closed-loop mechanism of “linguistic reasoning→latent prediction→path generation.” Its planning process relies less on external trajectory replay, instead conducting multi-step imagination and solution evaluation within latent space.

Research based on world models further expands the imaginative capacity of generative planning. DreamNav [83] simulates future paths in latent space to achieve language-guided planning under zero-shot conditions. Bar et al. [84] utilize conditional diffusion Transformers to learn vision–action correspondences, generating global path plans in unfamiliar environments through “imaginative trajectory simulation.”

From a functional perspective, high-level planning integrated with world models is equivalent to introducing an internal simulator at the policy level. Agents can predict future environmental changes based on experience and semantic understanding, form multiple path solutions through generative reasoning, and select optimal strategies using evaluation modules. Its advantages manifest in: (1) significantly reducing real environment interaction costs; (2) enhancing cross-task generalization capabilities; and (3) improving planning interpretability and forward-looking capacity. With the continuous fusion of generative models (such as Diffusion and VAE) with LLMs, this direction is poised to become a core research focus in embodied intelligent planning.

Paradigm comparison and application scenarios. Explicit reasoning relies on language-generated intermediate steps to provide transparent decision traces, facilitating debugging and human-in-the-loop interaction, but it often incurs higher latency and can be sensitive to prompt design. Implicit reasoning compresses decision-making into latent representations to enable efficient inference, making it suitable for resource-constrained or real-time systems, yet it typically sacrifices interpretability and makes failure attribution more difficult. World-model-based generative planning simulates candidate trajectories in latent space to support long-horizon and anticipatory decision-making, but it introduces higher system complexity and compute demand and may suffer from model mismatch. Table 3 summarizes representative methods for high-level planning.

### 3.4. Low-Level Motion Control

In LLM-empowered VLN systems, low-level motion planning is responsible for transforming language goals and visual perception into continuous, executable control signals, representing a critical component for achieving triple alignment among “language–perception–action”. Its core challenge lies in mapping semantically described goals in natural language into smooth, safe, and semantically consistent navigation actions within complex, dynamic environments. Unlike traditional embodied intelligence, large model-driven VLN not only relies on visual features and spatial constraints but also achieves integrated modeling of high-level goals and low-level control through the semantic understanding and generative capabilities of language models. With the development of reinforcement learning, imitation learning, and generative control, low-level control has gradually formed an evolutionary path from reactive behaviors to semantic generative control.

#### 3.4.1. Basic Control Strategies

Early VLN systems mostly relied on rule-based or reactive control methods, completing path following through predefined actions (e.g., move forward, turn, and stop). However, such methods exhibited limited performance in complex semantic scenarios, struggling to capture fine-grained correspondences between language and actions. With improvements in model capacity and cross-modal alignment capabilities, research has gradually shifted toward end-to-end joint modeling of language–vision–control, directly learning language-conditioned action generation policies in multimodal latent space.

Kåsene et al. [85] conducted a systematic comparison of control performance between low-level and panoramic action spaces, pointing out that continuous action modeling can more precisely align linguistic semantics with physical behavior, providing a theoretical foundation for fine-grained action generation. The NaviD system [46] adopts the Vicuna-7B language model and EVA-CLIP visual features, achieving smooth semantic navigation through parametric continuous control. SayNav [86] grounds high-level LLM planning by decomposing each reasoning step into short-range point-goal sub-tasks. These sub-tasks are then executed by a low-level planner as simple, discrete control commands, allowing SayNav to translate complex language-derived plans into a series of basic, actionable movements suitable for navigation in unfamiliar environments. Chen et al. [87] combined Grounded-SAM with Gemini-1.5-Pro, strengthening the “semantic-to-action” mapping precision through semantic segmentation and traversable area prediction. UAV-ON [88] introduces an open-world object-goal navigation benchmark for UAVs that centers on evaluating basic action-level control policies. By framing navigation as a sequence of discrete, high-level actions, the benchmark enables systematic analysis of decision-making behaviors under open-world conditions without involving closed-loop or dynamics-based control.

In terms of unified modeling, RT-2 [89] and OpenVLA [90] realize language-driven end-to-end action generation interfaces: both fuse visual and linguistic inputs into a shared latent representation, from which a language decoder directly generates executable action tokens, achieving integrated control from “understanding where to go” to “generating how to get there.” LaViRA [91] presents a unified translation mechanism for language–vision–action, transforming natural language instructions into continuous action sequences through sequential generation, achieving zero-shot navigation in unknown environments. It should be noted that although these models possess generative language interfaces and can directly output actions, their generation process remains based on single-step discriminative mapping, lacking latent modeling or sampling prediction of future trajectories. Therefore, this article classifies them under “end-to-end control strategy” rather than “generative control.”

Furthermore, some studies have begun introducing latent dynamics prediction mechanisms at the control layer. The Latent Dynamics Predictor (LDP) proposed in [92] can internally generate multi-step state trajectories to optimize control paths, marking low-level control’s transition from “explicit instruction response” toward “language-driven internal prediction,” laying the mechanistic foundation for subsequent generative control.

#### 3.4.2. Closed-Loop Control

In dynamic, unpredictable environments, low-level control requires adaptive and real-time correction capabilities. The closed-loop control mechanism is key to achieving this goal. Its core idea is to continuously perceive environmental changes during execution and instantly adjust action outputs through language or visual feedback, forming an adaptive loop of “perception–semantic–control”.

SkyVLN [93] couples vision–language navigation with a nonlinear model predictive controller (NMPC), enabling UAVs to execute closed-loop, dynamically feasible continuous control. By continually integrating visual–language cues into a feedback-driven optimization process, SkyVLN ensures safe and smooth motion through dense urban environments while respecting UAV dynamics. The UAV-VLN [94] implements end-to-end vision–language navigation on UAV platforms, dynamically correcting trajectories during flight through multimodal feedback loops, significantly enhancing stability and safety in complex environments. Narrate2Nav [95] introduces an implicit language feedback mechanism, enabling agents to adjust control commands in real-time based on natural language cues, thereby achieving more natural closed-loop interaction and semantic alignment in human-centric dynamic scenarios. CL-CoTNav [96] combines hierarchical chain-of-thought with closed-loop feedback mechanisms, achieving action self-correction through confidence-triggered re-reasoning cycles, further enhancing robustness and semantic consistency in zero-shot navigation. Zhang et al. [97], to address the challenges of window-level positioning difficulty and lack of prior maps in low-altitude terminal delivery, designed the LogisticsVLN framework. Centered on a lightweight multimodal large model, it substantially enhances the adaptability and robustness of multimodal large models in short-range, fine-grained dynamic outdoor scenes through a cascaded workflow design of request understanding, floor localization, and object exploration. Choutri et al. [98], to fill the gap in natural voice interfaces for UAV control, proposed an offline bilingual voice real-time control framework, building a HRI workflow centered on Vosk and Gemini. Through a synergistic mechanism of edge voice recognition, cloud-based semantic reasoning, and safe code generation, it significantly enhances the adaptability and robustness of HRI in multilingual, low-connectivity environments. Zhang et al. [99], addressing the problems of overly long paths and dense instructions in outdoor VLN, proposed the MMCNav framework. Centered on GPT-4o, it designs a multi-agent collaborative scheme adopting a working mode of macro instruction decomposition, multi-agent coordination, and dual-loop reflection and error correction, substantially enhancing the adaptability and robustness of multimodal large models in urban multi-agent collaborative navigation scenarios.

Closed-loop optimization reflects the transition of low-level control from static execution to dynamic self-regulation. Its advantage lies in enhanced robustness and environmental adaptability, providing a technical foundation for subsequent intelligent control that combines generative prediction with self-reflection mechanisms.

#### 3.4.3. Generative Control

With the deep integration of world models and generative models, low-level control in VLN has entered the generative modeling stage. Unlike traditional reactive control, generative control emphasizes “imagining” future trajectories in latent space, achieving more forward-looking and semantically consistent decisions through a “generate–evaluate–execute” closed loop.

DAgger [100] combines the imitation learning strategy with diffusion models, using expert demonstrations to guide diffusion policy updates, significantly alleviating drift and cumulative error issues in generative control. NavDP [101] generates multiple candidate navigation trajectories in latent space and introduces a critic mechanism for screening and optimization, utilizing privileged information during training to enhance generalization and stability, achieving zero fine-tuning migration from simulation to reality (Sim2Real). ComposableNav [102] employs composable diffusion models to generate continuous control sequences under linguistic conditions, enabling agents to achieve flexible, adaptable action generation in dynamic environments. Nunes et al. [103], to address the problem of manually writing control logic for aerial ad hoc networks, designed the FLUC framework. Based on Qwen 2.5 Coder, it builds a code generation workflow using a synergistic scheme of local offline inference, natural language-to-code translation, and ArduPilot execution, effectively enhancing system deployment adaptability and robustness in multilingual, offline scenarios.

Overall, generative control achieves a leap from “language-described navigation” to “language-driven imagination and generation.” By combining language models, world models, and diffusion generation mechanisms, agents can achieve semantically consistent, dynamically adaptable continuous control in complex scenarios, laying the foundation for true “cognition–behavior integration” embodied intelligence.

Category comparison and application scenarios. Basic control strategies are simpler and easier to stabilize, suiting discrete navigation settings, but struggle with fine-grained semantic constraints and accumulate errors over long horizons. Closed-loop control integrates online feedback for trajectory correction, improving robustness in dynamic environments, yet increases system overhead and requires careful tuning of perception–control interfaces. Generative control enables forward-looking, instruction-aligned trajectory synthesis, but remains computationally demanding and challenging for safety-critical deployment due to uncertainties in generated behaviors. Table 4 summarizes representative methods for low-level control, organized according to the evolutionary path from basic control to closed-loop control and generative control.

### 3.5. Discussion About LLM-Based VLN Systems

The various components of VLN are tightly coupled. To some extent, the VLN research framework closely resembles the traditional autonomous navigation research framework, namely “perception-planning/decision-control” [104]. The difference between the two lies in the fact that VLN involves HRI processes and requires an additional human instruction understanding module that is not present in traditional frameworks.

Essentially, the instruction understanding process in VLN tasks can be regarded as a semantic observation process, whose core function is to map natural language instructions into internal semantic states or goal constraints. This semantic state not only provides semantic priors for subsequent environmental perception but also establishes an interpretable task representation foundation for subsequent high-level planning and motion control stages.

Due to the polysemy, hierarchical nature, and context-dependency of human language, instruction understanding in VLN faces two major challenges: (1) cross-modal semantic alignment: how to map textual descriptions to specific targets or paths in visual scenes; and (2) generalization and interpretability: how to enable models to correctly understand instructions under unseen tasks or linguistic variations. With the rise of LLMs and VLMs, instruction understanding research has gradually shifted from template matching and sequence encoding to structured understanding based on semantic reasoning.

While traditional RNN-encoder-based methods are more friendly for resource-constrained devices in terms of efficiency and computational cost, their reasoning and interpretability capabilities are limited. LLM-based methods demonstrate stronger capabilities in semantic reasoning and knowledge generalization but incur higher computational overhead. Currently, relevant research is transitioning from traditional encoder models to semantic reasoning systems centered on LLMs. Moreover, modular architecture has become mainstream, employing lightweight encoders for front-end semantic extraction, while LLMs handle high-level logical reasoning, thereby achieving hierarchical and closed-loop language understanding. Language models no longer merely perform feature extraction but serve as high-level semantic interpreters, capable of generating structured task intents and reasoning chains that provide semantically consistent goal constraints for downstream modules.

Furthermore, the interaction logic of other components in the VLN framework largely aligns with traditional autonomous navigation pipelines, with only specific differences in the implementation of each module. For instance, the purpose of environmental perception is to construct spatial map representations consistent with environmental features for the agent, although such representations differ significantly from traditional map forms pursued by conventional SLAM technology. This process shares conceptual similarities with the human brain’s process of mapping the external physical world to form an internal “cognitive map” [105,106].

Overall, environmental feature learning, structured memory construction, and generative prediction correspond to the evolution stages from low-level to high-level in VLN agents’ map cognition. The ability to construct structured memory largely reflects the agent’s spatial-semantic consistency and environmental understanding capabilities over long time horizons. Regarding the generative prediction mechanism, which has emerged as a new direction in recent years, we believe its primary purpose in VLN is to build interpretable, predictable, and self-evolvable environmental cognition capabilities for agents through continuous optimization of “structured memory→generative prediction.” In fact, corresponding to the “cognitive map” concept, the neuroscience field also holds the “predictive map” viewpoint [107], which may provide a new reference for generative prediction. However, this evolutionary process has not yet formed a unified paradigm, and not all VLN technologies need to incorporate generative prediction mechanisms. For example, some researchers have attempted to build implicit structured memory using implicit neural fields [108], which also achieved promising results in VLN tasks. This issue deserves deeper exploration.

Additionally, the high-level planning component in the VLN framework aligns with the planning/decision-making component in existing “perception-planning/decision-control” pipelines, conducting decision reasoning for global/local path planning strategies based on environmental perception.

Overall, the development of high-level planning reflects the general trend of VLN evolving toward cognitive-level reasoning. The introduction of LLMs liberates high-level planning from traditional single mapping function limitations. No longer relying on black-box policy networks, it can generate interpretable decisions in the form of linguistic logic chains. Combined with world model-based generative planning mechanisms, agents can internally complete imagination and deduction of future states, demonstrating human-like cognitive reasoning capabilities. From early explicit logic chains to implicit latent modeling, and then to world model-based generative planning, the research paradigm is transitioning from “language-driven decision-making” to “cognitive generative reasoning.” This trend not only drives VLN’s transformation from “task execution” to “thinking and reasoning” but also provides new theoretical support and implementation pathways for future embodied intelligence.

Finally, there is the low-level motion planning component in the VLN framework, which aligns with the control component in the “perception–planning–control” pipeline.

Overall, LLM-driven VLN low-level control has evolved from rule-based behavior control to the semantic generative control stage. Control strategies have evolved from rule-based reactive mechanisms to adaptive generative control based on language and models. Through the combination of feedback optimization, imitation learning, and generative control, agents can maintain stability, robustness, and interpretability in dynamic environments, achieving end-to-end mapping from semantics to physics. Future VLN systems will no longer stop at “language-described navigation” but will achieve cognition-control integration from language understanding to action generation. Low-level motion planning will become the critical link connecting language understanding with embodied action, laying a solid foundation for embodied artificial intelligence to advance toward semantic autonomy.

## 4. Literature Review on Edge Deployment of LLM-Based VLN Systems

The evolution of large-scale pre-trained models has progressed from unimodal language processing to multimodal understanding and generation, driven fundamentally by continuous innovation in model architectures and training paradigms. Since the introduction of the Transformer architecture [109], pre-trained language models have largely coalesced into two dominant technical pathways represented by BERT [110] and GPT [111], focusing on language understanding and language generation, respectively. The rapid expansion of parameter scale, propelled by GPT-3, spurred the rise of prompt learning [112]. Subsequently, GPT-3.5 incorporated Reinforcement Learning from Human Feedback (RLHF) to achieve model alignment, catalyzing the emergence of ChatGPT(GPT-3.5) [113]. A significant leap was made by GPT-4, which achieved breakthroughs in cross-task reasoning and multilingual understanding [114].

Concurrently, developments in open-source and multimodal research advanced, with models like LLaMA [115] and Gemini [116] fostering community-driven exploration and modality fusion. BLIP-2 [117] proposed an efficient fusion paradigm of “frozen LLM + visual projector,” while Flamingo [118] utilized cross-attention mechanisms to demonstrate strong few-shot performance on visual question-answering tasks. By 2025, models such as GPT-5 [119], LLaMA-4 [120], Gemini 2.5, Qwen2.5-Omni [121], and the DeepSeek series [122,123] represent the latest advancements in multimodal unification and reasoning enhancement, signaling a shift in large model development from mere parameter scaling towards a comprehensive phase emphasizing alignment optimization, modality fusion, and reasoning augmentation.

For VLN systems, the navigation model must achieve high real-time performance and low energy consumption under constrained computational resources. However, LLM-based VLN models incur extremely high computational costs. Direct deployment on edge devices (e.g., mobile robots, unmanned vehicles, and IoT terminals) faces significant latency and energy consumption bottlenecks. Consequently, achieving efficient compression and deployment of LLMs has become a critical research focus for transitioning VLN technology from algorithmic research to practical application.

### 4.1. Pre-Deployment Optimization

Deploying LLMs on resource-constrained edge devices typically involves challenges such as limited storage, restricted bandwidth, and high inference computational costs. Therefore, systematic model compression and acceleration prior to deployment have become indispensable. Current research primarily focuses on core techniques, including quantization, pruning, knowledge distillation, and low-rank decomposition, often combined with various architectural and mechanistic optimizations. The goal is to significantly reduce inference costs while preserving model performance as much as possible. This section reviews these technical directions.

#### 4.1.1. Quantization

Quantization reduces model storage size and bandwidth requirements while enhancing the efficiency of matrix multiplication operations by mapping floating-point weights or activations to low-bit integers. In the context of LLMs, stable low-bit quantization remains challenging because activations often have a larger dynamic range, and the attention mechanism is particularly sensitive to numerical perturbations. Existing research can be broadly categorized into weight-only quantization and joint weight-activation quantization.

Weight-only quantization, which compresses only the model weights, is the easiest to deploy and generally has a relatively smaller impact on accuracy. The GPTQ method proposed by Frantar et al. [124] employs a one-shot, layer-wise quantization strategy based on approximate second-order information, enabling precise compression of GPT-series models to 3–4 bits without retraining, achieving high-precision Post-Training Quantization (PTQ). Lin et al. [125] proposed AWQ, which uses activation distribution to gauge weight importance, applying lower-bit quantization only to less critical weights, thereby maintaining stable performance in long-context and multi-task scenarios. Although these methods require a few activation samples for calibration, inference involves only integerized weights and does not require online handling of input-dependent activation distributions, making them highly practical and hardware-friendly for edge devices.

Joint weight-activation quantization further incorporates activations as compression targets, potentially offering higher acceleration ratios on bandwidth- and memory-constrained devices, but at a significantly increased implementation difficulty. MobileQuant, proposed by Tan et al. [126], establishes a complete integer-only inference pipeline for edge deployment. By simultaneously quantizing weights and activations to low bits and jointly optimizing weight transformations and activation quantization ranges, it reduces inference latency by approximately 20–50% on devices like Android, iOS, and Jetson. SmoothQuant [127] mitigates the challenge of outlier values in activations by collaboratively scaling weights and activations, effectively transferring the challenge of quantizing activations with outliers to the weights. This facilitates general W8A8 full-integer inference without significant accuracy loss. Yao et al. [128] proposed ZeroQuant, which employs a fine-grained group quantization strategy combined with layer-wise knowledge distillation and efficient system implementation for end-to-end PTQ of weights and activations, accelerating large-scale Transformer inference while maintaining stable accuracy at INT8, and even INT4 for some modules. Overall, joint quantization methods provide a critical foundation for achieving efficient, full-integer LLM inference.

#### 4.1.2. Pruning

Pruning reduces model size and FLOPs by removing redundant structures or weights, and can be classified into structured and unstructured pruning.

Structured pruning removes components at the granularity of channels, attention heads, or even entire layers. This approach is more hardware-friendly, as it directly reduces computational load and simplifies deployment. For example, the Sheared LLaMA [129], based on the LLaMA2-7B model, performs targeted structured pruning across multiple dimensions like network depth, number of attention heads, and FFN/hidden dimensions. Combined with a small amount of continued pre-training, it maintains performance superior to other open-source models of comparable size on multiple benchmarks, despite significantly reduced parameter count and training compute overhead.

Unstructured pruning sparsifies the model by selecting individual weights for removal. It can achieve higher sparsity levels for a given accuracy level, but its inference acceleration benefits are highly dependent on hardware support for sparse computations. SparseGPT [130] proposes a method for large-scale sparsification of GPT-series models in a one-shot, retraining-free manner, achieving sparsity levels of 50–60% with negligible perplexity increase. Movement Pruning [131] determines weight importance based on the magnitude and direction of weight updates during training, enabling more adaptive sparsification, particularly in transfer learning scenarios, and maintaining better downstream task performance at high sparsity levels. These two pruning techniques explore complementary paths for reducing the computational burden of LLMs from structural and parametric perspectives.

#### 4.1.3. Knowledge Distillation

Knowledge distillation transfers knowledge from a large, pre-trained teacher model to a smaller student model, enabling the compact student to approximate the performance of the larger teacher. It is a crucial technique for building efficient, lightweight models. Distillation can be categorized based on access to the teacher’s internal information.

White-box distillation allows access to the teacher’s internal information, such as attention distributions or hidden layer representations. This is the primary approach for distilling open-source models. MiniLLM [132] points out that traditional distillation using forward KL divergence can lead the student to overfit low-probability regions of the teacher’s distribution. It instead employs reverse KL divergence, more suitable for generative language models, along with an effective optimization strategy, creating a scalable distillation framework for various open-weight LLMs. MobileBERT [133] constructs a student architecture with bottleneck structures aligned to the teacher and uses a layer-wise distillation strategy, allowing the student to maintain task performance close to or even surpassing the teacher’s, despite significantly reduced parameters and inference latency.

Black-box distillation utilizes only the inputs and outputs of the teacher model, making it suitable for scenarios where the teacher is a proprietary API with inaccessible internal structures. Distilling Step-by-Step [134] leverages CoT annotations to distill the intermediate reasoning steps of a large model on multi-step reasoning tasks into a smaller model, significantly enhancing complex reasoning capabilities in compute-limited settings. Fine-tune-CoT [135] combines large-scale CoT supervision data with fine-tuning to transfer the complex reasoning abilities (e.g., logical reasoning) of hundred-billion-parameter teachers to student models with parameters in the hundred-million range, demonstrating the feasibility of data-driven reasoning distillation for building high-performance small models.

In summary, white-box and black-box distillation offer complementary compression pathways for open-source and proprietary LLMs, respectively, providing a scalable technical route for constructing high-performance small models.

#### 4.1.4. Low-Rank Decomposition

Low-rank decomposition leverages the redundancy within large weight matrices by approximating them as the product of smaller, low-rank matrices, thereby reducing parameter count and computational overhead. This method is particularly applicable to the linear transformations within attention and Feed-Forward Network (FFN) layers. ALBERT [136] reduces the overall parameter count significantly by factorizing the embedding matrix and sharing parameters across Transformer layers, maintaining or even improving performance on benchmarks while notably cutting storage and training costs. FWSVD [137] builds upon traditional Singular Value Decomposition (SVD) by incorporating weight importance metrics like Fisher information, applying weighted constraints to different singular vectors. This helps preserve task-loss-sensitive representations under the same rank constraint, leading to more stable performance of the compressed language model on various downstream tasks. Low-rank decomposition is complementary in principle to quantization, pruning, and distillation, potentially further increasing the overall compression ratio without altering the network topology.

#### 4.1.5. Other Methods

Beyond the primary compression strategies, various architectural and mechanistic optimizations have emerged as important supplements for improving inference efficiency on edge devices. These methods often reduce computational and memory burdens from a systems perspective without drastically cutting parameter counts.

Firstly, data preprocessing has proven crucial for enhancing the performance of compact LLMs. Through high-quality data selection, filtering, and synthesis, models can achieve stronger generalization without increasing size. Research, such as the work by Gunasekar et al. [138], demonstrated that even at the 1.3B parameter scale, using textbook-quality data and synthetic exercises can outperform larger models in tasks like code generation and commonsense reasoning.

Secondly, advanced positional encoding methods, like Rotary Position Embedding (RoPE) [139], enhance the model’s ability to handle long-range dependencies without substantially increasing computational overhead. RoPE, by applying a rotation Transformation in the complex plane based on token positions, incorporates relative positional information effectively and has become a standard component in models like LLaMA and Gemma.

Regarding attention structure, Multi-Query Attention (MQA) and Grouped-Query Attention (GQA) reduce the memory footprint and bandwidth demands of the Key£Value (KV) cache by sharing Key and Value projections across multiple heads (MQA) or grouping heads for sharing (GQA). Research [140] shows that GQA can reduce KV cache memory usage and access overhead by approximately 2–4 times compared to standard Multi-Head Attention, with minimal performance loss. This is particularly beneficial for mobile and edge devices with limited cache memory, making GQA a common feature in models like LLaMA and Qwen.

Finally, layer-wise scaling techniques improve the numerical stability of deep Transformer models by applying scaling factors to activations or weights across layers. Methods like DeLighT [141] employ block-level scaling across the network depth, making layers near the input shallower and narrower, and layers near the output deeper and wider. This allows for significantly increased network depth while maintaining training stability and inference performance. Such scaling methods can help mitigate numerical instability issues arising from operations like quantization or sparsification during post-compression fine-tuning, thereby improving convergence quality and final accuracy.

In summary, optimizations in data preprocessing, advanced positional encoding, GQA/MQA, and layer-wise scaling have become integral to the design of compact LLMs. They complement core compression/acceleration techniques like quantization, pruning, distillation, and low-rank decomposition, providing essential support for efficient inference in resource-constrained environments.

### 4.2. Runtime Optimization

#### 4.2.1. Software-Level Optimization

Software-level optimization is a critical pathway for enhancing the efficient operation of LLMs on edge devices, with research focusing primarily on cross-device collaborative computing, single-device resource scheduling, and execution framework optimization. These technologies collectively reduce inference latency and energy consumption at both algorithmic and systemic levels by eliminating redundant computations, improving resource scheduling strategies, and enhancing runtime system efficiency, thereby providing essential support for deployment in resource-constrained environments.

Cross-device collaborative computing aims to overcome the limitations of single-edge device computational capacity and storage by leveraging multi-device joint inference execution. Representative approaches include split inference and speculative decoding, which enhance overall efficiency from the perspectives of computational partitioning and interaction reduction, respectively. Split inference deploys different parts of a model across multiple computing nodes to achieve parallel execution across devices.

For instance, PETALS [142] employs a decentralized network architecture to distribute layers of a large Transformer across volunteer GPU nodes over the internet. Incorporated with fault-tolerant scheduling and load balancing, it maintains stable inference performance despite dynamic node availability and complex network conditions. Voltage [143] proposes a sequence-position-based computation partitioning method for Transformers across multiple edge devices. By reorganizing the self-attention computation flow within a single layer, it achieves near-linear acceleration through cross-device inference and significantly reduces end-to-end latency. Speculative decoding reduces the frequency of large model inference, thereby lowering interaction costs between cloud/remote and local devices. SpecTr [144] utilizes a lightweight model to draft a token sequence, which is then verified in parallel by a large model, substantially reducing the number of forward passes required by the large model. In scenarios where the large model resides in the cloud and a small model is deployed locally, this mechanism indirectly reduces cloud–edge interaction volume. Tabi [145] employs calibrated prediction confidence to determine whether a request or intermediate representation needs to be offloaded to a more powerful model, establishing a multi-tier inference system that compresses data transmission while maintaining accuracy. In summary, collaborative computing reorganizes the model inference pipeline across multiple devices, offering potential solutions for high throughput and low latency in edge scenarios constrained by bandwidth and computational resources.

Under single-device conditions, researchers optimize the inference graph execution flow from three directions, input reduction, early exiting, and dynamic resource allocation, aiming to maximize the local computational capability of the edge device. Input reduction methods decrease the computational burden by reducing the number of tokens processed during the forward pass. PoWER-BERT [146] progressively prunes less important intermediate token representations, effectively shortening the sequence length for subsequent layers. LLMLingua [147] adopts prompt compression, iteratively filtering prompts based on importance, significantly reducing input tokens and inference cost while preserving task performance. Early exiting attaches internal classifiers to intermediate Transformer layers, allowing inference to terminate early if predictions reach sufficient confidence. PABEE [148] introduces a “patience mechanism,” stopping inference when predictions stabilize across consecutive layers, saving substantial subsequent computations. MPEE [149] unifies vertical (inter-layer) and horizontal (token-level) exiting strategies, enhancing flexibility and achieving better performance–efficiency trade-offs across varying input lengths and tasks. Dynamic resource allocation improves overall execution efficiency by rescheduling data and operator execution among the device’s CPU, GPU, and storage units. STI [150] partitions model parameters into shards of varying importance, combined with prefetch caching and elastic pipelining, enabling efficient Transformer inference under severely limited device memory. FlexGen [151] proposes a hybrid memory management strategy involving GPU, CPU, and disk. By optimizing data access patterns and I/O scheduling via linear programming, it facilitates high-throughput batch inference for large models, offering insights for resource-constrained devices. These methods reduce local computational load from various angles, input processing, inference path, and storage scheduling, enabling edge devices to support efficient inference for relatively large models even in standalone settings.

Framework-level optimization focuses on building lightweight, high-efficiency execution engines to support stable, low-latency operation of large models on edge devices. ExecuTorch [152], as PyTorch’s unified edge inference framework, significantly reduces runtime overhead on mobile and embedded platforms through graph-level optimizations, operator customization, and execution plan generation. DNNFusion [153] employs advanced operator fusion techniques to merge multiple operators into efficient compound operators, reducing memory access and kernel invocation overhead. SmartMem [154] analyzes layout Transformation patterns in model execution graphs to automatically eliminate redundant data format conversions, markedly decreasing memory access costs during on-device execution. Regarding runtime systems, PagedAttention [155] draws inspiration from virtual memory management. It partitions the KV cache into reusable memory blocks, enabling sharing across requests. Coupled with optimized GPU kernels and quantization support, it improves throughput and memory utilization, showing promising scalability for memory-constrained edge GPU scenarios.

Software-level optimizations form a relatively comprehensive technical stack encompassing cross-device collaboration, intra-device scheduling, and execution frameworks, complementing hardware optimizations and model compression techniques. Future efficient LLM deployment will increasingly rely on hardware–software co-design, involving joint optimization of runtime scheduling, data layout, memory hierarchy, and model architecture to continuously reduce inference latency and energy consumption in edge environments, laying the groundwork for the widespread adoption of large models in resource-constrained settings.

#### 4.2.2. Hardware-Level Optimization

Hardware-level optimization provides the foundational computational support for deploying LLMs on edge devices from an architectural perspective, primarily involving the analysis of the roles, performance characteristics, and limitations of CPUs, GPUs, and NPUs during inference. As model parameters grow, effectively leveraging hardware potential under strict power and computational constraints in edge scenarios becomes a central challenge for LLM deployment.

First, the CPU, as a general-purpose processing unit, remains relevant for lightweight model inference due to its high flexibility and mature software ecosystem. Recent quantization methods tailored for edge scenarios have significantly boosted LLM inference efficiency on CPUs. For example, Shen et al. [156] proposed an activation-guided quantization strategy that co-adjusts quantization intervals for weights and activations, achieving over 2× acceleration for LLM inference on various edge devices. The Intel i9-13900K, paired with AQLM [157], demonstrates efficient execution of models like LLaMA-2, indicating that advanced quantization can unlock the potential of low-precision integer computation on CPUs. However, CPUs still face performance bottlenecks when handling large-scale Transformer inference due to limited parallelism and matrix multiplication capabilities. Consequently, modern Systems on Chip (SoCs) commonly adopt heterogeneous architectures integrating CPU, GPU, and NPU (e.g., Apple A/M series [158] and Google Tensor G series [159]) to enhance overall inference efficiency through collaborative task distribution.

Second, GPUs, with their powerful parallel matrix computation capabilities, are primary accelerators for medium to large LLMs on the edge. Represented by platforms like the NVIDIA Jetson series (e.g., AGX Orin [160]), these low-power GPU platforms can execute relatively large Transformer models within limited power budgets, supported by features like Tensor Cores and high memory bandwidth. Studies have explored offloading parts or whole models to such accelerated platforms to assess the feasibility of edge-side LLM inference (e.g., Yuan et al. [161]’s tested on Orin NX). Meanwhile, as sensitivity to energy efficiency and cost in edge scenarios increases, cloud–edge collaborative inference schemes are gaining traction. Zhang et al. [162] demonstrated that dynamically partitioning computational tasks between cloud and edge over wireless networks can reduce generative LLM inference latency without significantly increasing energy consumption, highlighting the trade-offs between GPU power and edge responsiveness. Furthermore, task partitioning and synchronization among GPU, CPU, and NPU in heterogeneous architectures add complexity to system design.

Finally, NPUs are specifically designed for neural network inference, achieving high energy efficiency through highly parallel low-precision integer computation (e.g., INT8). They are increasingly important inference engines in mobile and edge devices. For instance, the Apple Neural Engine (M2 Ultra) [163] and Snapdragon AI Engine (8 Gen 3) [164] incorporate higher compute density, lower power consumption, and richer acceleration instructions, providing the hardware foundation for running more complex models on-device. However, current research indicates that NPUs still have limitations in operator coverage, software stack maturity, and support for emerging LLM architectures. MobileLLM [165] suggests that deep modifications and operator simplification are necessary to adapt sub-billion parameter models to mobile devices, reflecting the existing gaps in NPU support for new LLM architectures. In practice, as some models or operators cannot be fully mapped to NPUs, CPUs or GPUs are often required to assist in the inference pipeline, further complicating scheduling in heterogeneous systems.

In summary, CPUs offer generality and flexibility, GPUs provide core parallel computing power, and NPUs excel in energy efficiency. Together, they form the hardware foundation for on-device LLM inference. Combined with software-level optimizations (e.g., quantization, operator fusion, and cache management) and hardware–software co-design, they provide crucial support for the efficient deployment of large models in resource-constrained environments.

#### 4.2.3. Hardware–Software Co-Design

Hardware–software co-design is a cross-layer optimization paradigm that establishes tight coupling between model algorithms and hardware micro-architecture to enable efficient inference of LLMs on resource-constrained edge devices. Unlike isolated model compression or hardware acceleration, co-design emphasizes the bidirectional adaptation of algorithm-data flow-operator patterns-hardware execution units, primarily manifested in hardware-aware sparsification and hardware-optimized arithmetic formats.

Hardware-aware sparsification creates structured, predictable, and stable sparsity patterns within the model, enabling the underlying hardware to efficiently map matrix and attention computations in Transformer designs. In such co-designed schemes, sparsification is not merely a model compression technique but an integral part of the design coordinated with the hardware’s memory hierarchy, network bandwidth, and parallel execution units. In the direction of custom ASIC accelerators, Wang et al. [166] cascaded token pruning and attention head pruning to reduce the effective density of attention matrices, allowing the sparse patterns to be efficiently parsed within the hardware pipeline, thereby reducing memory access and computational overhead. Sanger [167] integrates a dynamic structured sparsity mechanism with a reconfigurable accelerator architecture, achieving adaptability to different tasks and input conditions and demonstrating significant acceleration under sparse attention scenarios. In Processing-In-Memory (PIM/CIM) architectures, hardware-aware sparsification can notably reduce energy overhead associated with data movement. For instance, TransPIM [168] leverages high-bandwidth PIM structures to construct token-level data flows, mapping sparse attention into more regular memory access patterns, thus lowering communication costs. X-Former [169] co-designs the sparse execution of Transformer modules with NVM/CMOS hybrid memory arrays, achieving higher energy-efficient sparse inference by coupling with a software-side attention engine. Overall, ASIC solutions often excel in throughput, while PIM architectures hold a clear advantage in energy efficiency by reducing off-chip access, both showcasing the potential of hardware-aware sparsification for edge LLM inference.

Hardware-optimized arithmetic formats aim to achieve higher energy efficiency and smaller area overhead for hardware executing deep models by reducing bit-width, adjusting encoding structures, or providing dynamically adaptable floating-point representations, while maintaining model accuracy at lower precision. Low-bitwidth fixed-point formats are a core direction. GOBO [170] compresses the attention module to a 3-bit parameter representation, combined with specific encoding strategies, significantly reducing hardware area and inference energy. Mokey [171] proposes a general fixed-point quantization framework enabling Transformer models without quantization-aware training to perform inference directly at 4-bit, improving the energy efficiency utilization of hardware accelerators. Dynamic floating-point encoding methods adjust the numerical range at runtime to mitigate overflow and precision loss at low bit-widths. AdaptivFloat [172] dynamically adjusts the exponent bias at the tensor level, allowing accelerators to better adapt to numerical distributions while maintaining very low bit-widths. The Flint format in ANT [173] further supports mixed integer and floating-point encoding, enabling accelerators to flexibly switch between arithmetic modes to meet cross-task precision requirements and energy constraints.

In summary, fixed low-bitwidth formats (e.g., 3-bit or 4-bit fixed-point) are more suitable for specialized accelerators with stable structures and high energy efficiency sensitivity, while dynamic/hybrid numerical formats are better for generalized accelerator systems requiring high precision or cross-task adaptability. The combination of both directions will provide a more flexible numerical foundation for future efficient LLM inference at the edge.

### 4.3. VLN Edge Deployment Cases

As LLM-based VLN systems transition from laboratory settings to real-world deployment on platforms like mobile robots, drones, and embedded systems, achieving stable and efficient multimodal reasoning under constraints of computation, power, and storage becomes a core challenge. Recent research has explored this along two dimensions: model-side compression/architectural optimization (corresponding to Category 4.1 techniques) and system-level inference pipeline restructuring (corresponding to Category 4.2 techniques), gradually forming representative edge deployment paradigms. Table 5 summarizes representative efficient and edge-deployable VLN/LLM-based navigation systems across these two dimensions.

In the direction of model-side lightweighting, TinyVLA [174] compresses vision–language–action modeling to a scale of 70 M–1.3 B parameters and replaces traditional autoregressive decoding with diffusion-based parallel action generation, significantly reducing inference latency while maintaining expressiveness for complex manipulation tasks, exemplifying the Category A approach of structural simplification. EdgeVLA [175] specifically targets the bottleneck of action decoding. By constructing a fully non-autoregressive action generation structure, the model outputs control sequences in one shot, achieving orders-of-magnitude acceleration on edge GPUs, demonstrating the critical role of architectural redesign for low-power devices. Lite VLA [176] targets extreme low-power, CPU-only platforms. Combining a small VLA model, LoRA-based parameter-efficient fine-tuning, and 4-bit quantization enables the system to run independently on micro-devices like Raspberry Pi. Coupled with an action chunking strategy to improve control stability under low inference frequency, it represents a co-designed approach combining compression and control mechanisms. The Edge LLMs work by Gurunathan et al. [177] constructs a multimodal model family comprising a lightweight visual encoder and a multi-scale language model, capable of stable, real-time environmental understanding on various devices like mobile SoCs and Jetson, providing a general, reusable model base for subsequent on-device VLN systems. Furthermore, GRaD-Nav++ [178] extends the lightweight VLA concept to aerial robots. Integrating visual–language perception, Gaussian splatting mapping, and differentiable dynamics into a lightweight framework capable of running entirely on onboard compute, and enhancing cross-task generalization via a Mixture of Experts (MoE) action head, demonstrates the deployability of the model-side lightweighting path for both ground and aerial scenarios. These methods illustrate the practical application of Category A techniques for edge deployment from various angles: model size reduction, action structure redesign, quantization, parameter-efficient fine-tuning, and lightweight multimodal backbones.

Complementing model-side optimizations, system-level inference pipeline restructuring significantly reduces the on-device computational burden by reorganizing visual input, historical memory, reasoning paths, and execution modes, without necessarily drastically compressing model size. EfficientNav [179] introduces a retrievable navigation memory cache, compressing long-context reasoning into manageable semantic snippets, reducing KV cache and prefill overhead, allowing medium-sized models to accomplish long-horizon navigation tasks on Jetson Orin. SINGER [180] constructs an end-to-end lightweight closed-loop pipeline from “vision–language–control.” It uses semantic heatmaps derived from CLIPSeg instead of high-dimensional visual inputs and a small control network for high-frequency flight control, enabling drones to perform language-guided navigation in real-time (12–20 Hz) on onboard computers without SLAM or large models, representing a typical optimization via pipeline rearrangement and representation substitution.

PanoGen++ [181] addresses the challenge from an environment generation perspective. By building a domain-adaptive panoramic semantic environment generation model, it allows the VLN system to maintain stable planning even lacking real-time complex visual input, reducing reliance on heavy visual encoders and large multimodal models during operation. The LLM-based Parameter-Efficient Action Planning (PEAP) [182] utilizes structured reasoning templates (e.g., CoT-style planning) to equip small and medium-sized language models with complex planning capabilities, alleviating the reliance on large model inference without scaling up. VLN-PETL [183] approaches from a transfer learning angle, using parameter-efficient fine-tuning to build modular, reusable skill units, enabling models to adapt quickly to new environments and reducing online computation needs. Building on this, VL-Nav [184] demonstrates the potential of system-level optimization for on-device mobile robots: integrating pixel-level semantic features from open-vocabulary vision–language models with a frontier-based semantic-driven goal selection strategy achieves 30 Hz real-time zero-shot navigation on Jetson Orin NX, significantly reducing dependence on online large model inference.

ClipRover [185] constructs a language relevance database and a modular reasoning pipeline, enabling zero-shot target exploration using monocular vision alone, which markedly reduces online multimodal computation overhead and allows execution of semantic navigation and active exploration tasks on resource-constrained UGV platforms. These system-level methods exemplify the core idea of Category B techniques: through pipeline restructuring, semantic representation compression, retrievable memory, structured planning, and skill modularization, the overall reasoning burden is significantly reduced, maintaining real-time performance and stability even on constrained hardware.

## 5. Implementation Requirements and Evaluation Protocols

In VLN research, the continuous improvement of model performance relies not only on advances in algorithm design and multimodal fusion mechanisms but also on the availability of high-quality data resources and systematic evaluation frameworks. Datasets determine the semantic space in which models learn and generalize, while evaluation metrics define the dimensions of comparison and guide methodological development. The former answers “what to learn and under what scenarios,” whereas the latter addresses “how well the model learns and whether it can generalize.” Therefore, building a comprehensive data and evaluation ecosystem is essential for advancing VLN from laboratory prototypes to real embodied-intelligence applications.

Overall, the research foundation of VLN comprises three key components: datasets, simulation or reconstructed environments, and evaluation metrics. Datasets specify task definitions and language–action mappings; simulation and reconstructed environments provide interaction capabilities and physical grounding; and evaluation metrics offer standardized criteria for cross-method comparison. Together, these elements form the experimental and assessment framework that supports the development of VLN.

### 5.1. Datasets

In VLN, high-quality datasets play a fundamental role in advancing model performance and semantic generalization. Task-oriented datasets provide explicit learning objectives and evaluation standards, whereas environment and simulation datasets supply diverse and controllable 3D scenes for embodied perception and interaction. These two dataset types are mutually dependent and jointly support the rapid development of the VLN research community. Since 2018, the VLN data ecosystem has undergone parallel evolution in task design and environment construction, gradually forming a research trajectory centered on task-driven navigation.

The earliest Room-to-Room (R2R) dataset [4], built on the Matterport3D environment, defines discrete paths from a start point to a goal location accompanied by natural-language instructions, establishing the foundational paradigm for VLN. RoomNav [186], developed on the House3D platform, introduces semantic goal-oriented navigation for the first time. R4R [187] extends R2R by increasing path length and instruction complexity and proposes the Coverage weighted by Length Score (CLS). RxR [188] incorporates multilingual instructions and dense temporal alignment in MP3D, enabling cross-lingual navigation. CVDN [189] expands VLN to multi-turn dialog-based interaction, allowing agents to adjust navigation trajectories based on human feedback.

In continuous control and embodied-interaction settings, VLN-CE [190] leverages the Habitat platform to evaluate navigation in continuous action spaces, narrowing the gap between simulation and the real world. REVERIE [5] integrates target recognition with navigation, while ALFRED [191], built on AI2-THOR, introduces object manipulation, driving VLN toward embodied semantic reasoning. SOON [192], HM3D [193], and HM3D-SEM [194] enhance semantic reasoning and scene understanding through semantic-level goals and open-environment exploration. In outdoor navigation, Talk2Nav [195] employs Google Street View (GSV) to construct large-scale urban visual–language navigation tasks, supporting long-distance street-level navigation. Most recently, DDN [6] emphasizes demand-driven language understanding and goal generation, enabling agents to execute complex behavior planning based on natural instructions, and marking a shift in VLN from “path following” toward “intent understanding.”

Overall, as summarized in Table 6, task datasets answer the question of “what the agent should accomplish,” forming the core basis for model learning objectives and performance evaluation. With the increasing scale, semantic richness, and interaction complexity of available datasets, the VLN data landscape has evolved from static path-following tasks toward dynamic, embodied, and multimodal tasks, providing a solid data foundation for the development of large-model-driven embodied agents.

### 5.2. Simulation and Reconstructed Environments

Simulation and reconstructed environments provide VLN agents with high-fidelity visual inputs, physical interaction capabilities, and spatial semantics, forming a critical foundation that bridges language understanding and action execution. The integration of these environments enables agents to learn and validate complex tasks within controllable and repeatable 3D settings.

For indoor environments, Matterport3D (MP3D) [196], introduced by Chang et al. in 2017, includes 90 buildings and approximately 10,800 panoramic RGB-D images and serves as the primary environment for tasks such as R2R, R4R, and RxR. House3D [186], introduced in 2018, provides over 45,000 procedurally generated indoor layouts with room labels, object categories, and customizable agent viewpoints. As a lightweight but large-scale environment, it supports tasks such as RoomNav and serves as an early platform for semantic goal-driven embodied navigation. Compared with MP3D, House3D offers greater diversity and controllability but lacks photorealism and real-world structural fidelity, which limits its effectiveness for sim-to-real research. Gibson [197] provides over 1400 high-quality indoor scanned scenes, and its extended version, iGibson, incorporates a physics engine and object-state variations, making it widely used in embodied manipulation and Sim2Real transfer research. HM3D and HM3D-SEM, released by Meta AI between 2021 and 2023, expand this further by offering large-scale building-level scans and fine-grained semantic annotations, representing the most extensive and semantically rich indoor reconstruction datasets to date.

In terms of simulation platforms, Habitat [198] is currently the most widely used 3D simulation framework. It supports MP3D, Gibson, HM3D, and other environments, accommodates multimodal inputs, and enables continuous action control. Habitat serves as the unified platform for tasks such as VLN-CE, SOON, and HM3D-NAV. AI2-THOR [199], emphasizing interactive physical simulation, supports object grasping, switching, and placement operations, and is the primary platform for “navigation + manipulation” tasks such as ALFRED and DDN. RoboTHOR [200] further provides paired simulated and real indoor scenes, enabling systematic evaluation of sim-to-real transfer in embodied navigation and object-search scenarios.

For outdoor environments, GSV and StreetNav [201] represent two major lines of simulation-based exploration. GSV is suitable for offline multimodal learning using large-scale street-view imagery, whereas StreetNav is designed for real-time navigation and human–agent interaction using sequential video streams. In addition, AirSim [202] provides photorealistic outdoor and urban scenes with realistic UAV dynamics, offering a controllable platform for studying continuous-control aerial navigation, obstacle avoidance, and vision-guided UAV behavior.

Overall, as summarized in Table 7a, simulation and reconstructed environments answer the question of “where the agent can operate,” providing VLN models with reproducible and scalable experimental conditions. High-fidelity 3D reconstructions and interactive simulation platforms enable research to advance from static perceptual understanding to dynamic policy learning and offer essential support for integrating multimodal LLMs with embodied agents. To further support practical platform selection under a unified criterion, Table 7b provides an at-a-glance comparison of representative simulators/scene sources along consistent dimensions, including domain, scene source, interaction/physics support, and Sim2Real relevance.

### 5.3. Evaluation Metrics

A scientific and well-structured evaluation framework is essential for assessing VLN model performance and comparing different methods. Such a framework measures an agent’s navigation and instruction-following ability from multiple dimensions—including path execution quality, task success, and semantic consistency—thereby providing a comprehensive view of model behavior under natural-language commands. Existing evaluation protocols can be broadly categorized into path-level metrics and semantic-level metrics.

#### 5.3.1. Path-Level Metrics

The VLN community commonly adopts five core path-level metrics:

- Success Rate (SR) [203]: The proportion of episodes in which the agent reaches the target within an acceptable error tolerance;
- Trajectory Length (TL) [204]: The average length of the agent’s executed trajectory, reflecting path efficiency;
- Success weighted by Path Length (SPL) [203]: A composite metric that accounts for both success and path optimality, and is widely considered the most representative performance indicator;
- Oracle Success Rate (OSR) [4]: An episode is counted as successful if the agent enters the goal region at any timestep, regardless of its final stopping action;
- Navigation Error (NE) [4]: The shortest-path distance between the agent’s final position and the goal location.

As summarized in Table 8, SR, SPL, and NE form the primary evaluation backbone for navigation performance, while TL and OSR are often used to analyze path efficiency and stopping/exploration behavior. Path-level metrics are effective for evaluating whether an agent can reach a specified goal and how efficiently it navigates. In particular, SR provides a coarse measure of task completion under an error tolerance, while SPL incorporates both success and path efficiency and is therefore widely adopted for benchmarking. However, these metrics may be insufficient to reflect instruction faithfulness: an agent can achieve high SR/SPL while missing intermediate landmarks or deviating from fine-grained language constraints. Moreover, OSR can overestimate performance for policies that transiently enter the goal region without reliably stopping, and TL/NE do not capture whether the trajectory is semantically consistent with the instruction. Therefore, path-level metrics are best interpreted as outcome/efficiency indicators, and should be complemented by process- and semantics-aware metrics when the task involves complex language grounding.

#### 5.3.2. Semantic- and Process-Level Metrics

As VLN tasks evolve from simple path execution to semantic understanding and interactive control, path-level metrics alone are no longer sufficient to capture the full spectrum of agent capabilities. To address this, researchers have proposed a variety of semantic consistency and interaction-performance metrics. Normalized Dynamic Time Warping (NDTW) and Success weighted Dynamic Time Warping (SDTW) [205] measure the temporal and spatial alignment between predicted and reference trajectories. Coverage weighted Length Score (CLS) [187] evaluates the consistency between the navigation path and the language description by incorporating both path coverage and length constraints. Goal Progress (GP) quantifies the agent’s average progress toward the target when the goal is not reached. Combined metrics such as SR-CLS jointly measure success in both localization and semantic recognition, particularly in tasks such as REVERIE and ALFRED.

In multi-turn interactive settings (e.g., CVDN), additional metrics have been introduced, including Dialog Success Rate (DSR), Response Appropriateness (RA), and Dialog Efficiency (DE), which assess language comprehension and collaborative performance during human–agent interactions.

Semantic- and process-level metrics provide a finer-grained assessment of trajectory quality and instruction compliance. Metrics such as NDTW/SDTW quantify alignment to a reference trajectory and are particularly informative in path-following settings where a canonical route is available. Nevertheless, in goal-oriented or exploration-heavy tasks where multiple trajectories can be valid, strict trajectory-matching may be less appropriate and can favor dataset-specific biases. CLS improves language–trajectory consistency evaluation by incorporating coverage and length constraints, yet it remains an indirect proxy for semantic grounding and may not fully capture whether specific linguistic constraints are satisfied. GP is useful for diagnosing partial progress in failure cases, but should not be interpreted as success or instruction compliance. Overall, these metrics complement SR/SPL by revealing how an agent navigates, but their applicability depends on whether reference paths or interaction protocols are well-defined. As summarized in Table 9, the VLN evaluation framework is shifting from purely outcome-driven metrics toward more semantic and process-oriented assessments, providing a more comprehensive basis for model comparison in complex multimodal interactive environments.

Practical guideline. For path-following VLN, we recommend reporting SPL (or SR), together with NDTW/SDTW (and/or CLS), to jointly capture success, efficiency, and route compliance. For goal-oriented navigation/object search, outcome metrics (SR/OSR) should be paired with efficiency and robustness measures (e.g., TL/steps, NE, and collision rate when available), since a unique reference trajectory may not exist. For interactive/dialog VLN, dialog-centric metrics (DSR/RA/DE) should be reported alongside navigation success to reflect collaborative effectiveness. For real-world/Sim2Real evaluation, safety- and reliability-related metrics (e.g., collision/near-miss statistics, recovery frequency, and latency proxies) become critical complements to geometric success.

## 6. Challenges and Future Trends

LLM-based VLN is driving embodied agents toward an integrated “language–perception–action” form of intelligence. This paper has systematically reviewed the evolution of VLN tasks, core system components, and datasets and evaluation protocols, as well as recent progress in edge-oriented deployment. Overall, although LLM-enabled VLN demonstrates substantial potential, the transition from research prototypes to real-world deployment remains challenged at multiple levels. These issues can be broadly categorized into capability-level limitations and ecosystem-level constraints. Correspondingly, future research should focus on enhancing the intelligent capabilities of VLN systems and strengthening the surrounding research ecosystem.

### 6.1. Major Challenges

#### 6.1.1. Capability-Level Constraints

Current VLN systems empowered by LLMs still face several fundamental capability constraints:

- Insufficient semantic reasoning and logical planning: Although LLMs have achieved significant progress in language understanding and contextual modeling, their reasoning is still dominated by statistical correlations rather than structured logic or causal inference. As a result, they struggle with multi-step commands, nested conditions, and implicit constraints, limiting long-horizon planning and cross-scene generalization;
- Limited spatial understanding and lack of world models: Existing VLMs mainly capture local spatial relations but fail to systematically model dynamic scenes, object interactions, and physical constraints. Without predictive world models, agents lack anticipatory spatial cognition and physical reasoning, which is essential for real-world navigation [206,207];
- Hallucination and robustness issues: LLMs and VLMs often produce hallucinations, such as incorrect semantic descriptions or object predictions, which can lead to invalid or unsafe navigation decisions. The lack of cross-modal consistency checking and external knowledge validation is a key cause of this problem [208];
- Real-time constraints and computational bottlenecks: High inference latency and energy consumption of LLMs create challenges for deployment on mobile robots and edge devices. Balancing accuracy, latency, and energy efficiency remains a major obstacle for embodied navigation systems.

#### 6.1.2. Ecosystem-Level Constraints

In addition to capability limitations, ecosystem-level issues also hinder the development and real-world deployment of VLN:

- Insufficient dataset and task diversity: Most existing VLN datasets (e.g., R2R, RxR, and VLN-CE) are limited to static indoor scenes and lack multilingual, multimodal, and dynamic interaction data, resulting in poor generalization to real-world environments;
- Incomplete evaluation systems and benchmark standards: Current metrics such as SR, SPL, and nDTW focus primarily on geometric indicators and fail to capture semantic understanding, reasoning quality, and interaction efficiency, making it difficult to compare models across tasks and platforms;
- Challenges in Sim2Real transfer: Simulation platforms (e.g., Habitat and AI2-THOR) simplify physical properties such as friction, inertia, and collisions, leading to significant performance degradation when models are deployed on real robots. The lack of standardized simulation–real-world integrated frameworks further limits engineering-level deployment [209].

#### 6.1.3. Deployment-Level Constraints

Despite rapid progress in simulation, bringing LLM-enabled VLN to practical deployment requires addressing several challenges beyond algorithm design:

- Deployment toolchains and runtime constraints: Integrating LLM/VLM components with robotics stacks demands hardware-aware compilation, optimized inference runtimes, and careful management of memory-intensive modules (e.g., KV caches) to meet strict latency budgets;
- Compression-induced failure modes: Quantization, pruning, and distillation are often necessary for edge deployment, yet they may degrade cross-modal alignment and long-horizon reasoning, reducing instruction faithfulness and, in some cases, increasing instability or hallucination-like behaviors under distribution shifts.
- Edge resource limitations and system coordination: Compute, memory, energy, and bandwidth constraints demand dynamic scheduling across perception, mapping, planning, and control modules, potentially requiring edge–cloud partitioning with robust communication to ensure reliability and safety in closed-loop execution for real robots.

### 6.2. Future Directions

#### 6.2.1. Capability Enhancement

Promising directions for improving the capabilities of LLM-enabled VLN include:

- Knowledge augmentation and semantic–logical reasoning integration: Incorporating knowledge graphs, structured reasoning, and causal inference can enhance logical consistency and improve complex instruction understanding [206];
- Unified multimodal representation and world-model construction: Integrating vision, language, touch, and other sensory modalities into a unified representation space, and combining it with predictive world models, can help agents transition from semantic alignment to physical understanding [207];
- Lightweight architectures and efficient inference optimization: Techniques such as Mixture-of-Experts (MoE), LoRA, model quantization (GPTQ and QLoRA), and cloud–edge collaborative inference can reduce latency and energy consumption;
- Robustness and safety enhancement: Multimodal consistency checking, external knowledge validation, and reinforcement learning with human feedback (RLHF) can mitigate hallucinations and improve system stability, safety, and interpretability [208].
- Sim2Real gap mitigation for deployable VLN: Leveraging language/semantic representations as more domain-invariant abstractions, together with domain randomization and real-world calibration, paired sim–real evaluation protocols, and robustness-oriented policy learning, can reduce appearance/dynamics mismatches and improve transfer to physical robots;
- Multi-agent collaboration and communication-efficient reasoning: For multi-robot VLN, future work should account for communication overhead (token/bandwidth/latency and synchronization frequency), develop centralized/decentralized collaborative reasoning with consensus/conflict resolution, and leverage shared semantic memories/maps plus compressed, event-triggered messaging (e.g., structured {goal, landmark, action, and confidence}) to improve scalability; corresponding benchmarks should jointly evaluate task success and communication cost.

#### 6.2.2. Ecosystem Development

Advancing the VLN ecosystem requires coordinated improvements in data, evaluation, and deployment frameworks:

- Construction of diverse and large-scale datasets: Future datasets should include multilingual, multimodal, and interactive navigation tasks to better reflect real-world complexity;
- Development of unified and reproducible benchmarks: Standardized evaluation frameworks can more systematically assess semantic understanding, reasoning depth, and execution quality;
- Integrated Sim2Real and Real2Sim development frameworks: Realistic simulation of sensor noise, physical disturbances, and robot-specific constraints can enable closed-loop training–validation–deployment pipelines [209];
- Multi-task unified frameworks: Models capable of jointly handling perception, reasoning, interaction, and control can promote VLN toward more general-purpose embodied agents [29,210,211].

### 6.3. Comprehensive Outlook

In summary, LLM-enabled VLN is currently undergoing a critical transition from modality fusion to cognitive integration. Future progress will require a dual-driving framework that simultaneously advances intelligent capability and strengthens the supporting research ecosystem. The former determines the depth of reasoning and the level of autonomy achievable by VLN systems, while the latter governs the transferability and practical deployability of research outcomes. By incorporating world models, enhancing knowledge-based reasoning, improving datasets and evaluation protocols, and enabling efficient deployment, VLN systems may progress from language-level interpretation toward physical-world understanding, ultimately moving toward an era of explainable, generalizable, and deployable embodied navigation [212].

## 7. Conclusions

This review provides a comprehensive synthesis of recent advances in LLM-enabled VLN across four key components: instruction understanding, environmental perception, high-level planning, and low-level motion control. The analysis highlights a clear shift in VLN from traditional modality matching toward a more integrated pipeline that spans semantic reasoning, cognitive planning, and behavior generation. Leveraging the strengths of LLMs in semantic understanding, cross-modal alignment, and knowledge generalization, VLN systems are gradually forming a unified semantic–cognitive–control framework that enhances generalization, interpretability, and task adaptability in previously unseen or dynamic environments.

In addition, this paper summarizes emerging techniques for edge deployment—including quantization, pruning, distillation, and lightweight multimodal fusion—and emphasizes the importance of real-time performance, energy efficiency, safety, and Sim2Real readiness for practical adoption. Overall, LLM-driven VLN is undergoing a critical transition from multimodal fusion toward deeper cognitive integration. Future progress is expected to focus on unified decision frameworks grounded in world models, tighter coupling between language, vision, and spatial memory, multi-agent collaborative navigation, and lightweight VLN systems that are interpretable and deployable. With continued advances in foundation models and embodied AI, VLN is poised to deliver broader value in real-world applications.

## Figures and Tables

**Table 1:** Representative methods for instruction understanding in VLN.

Category	Methods	Year	Contributions
Instruction Semantic Encoding	Speaker-Follower [24]	2018	Encodes instruction sequences with LSTM and aligns them with trajectories through data augmentation.
	RCM [25]	2019	Reinforces cross-modal semantic alignment using matching rewards to improve global language–vision consistency.
	DDN [6]	2023	Extracts attribute-level semantics via LLM and aligns them with CLIP embeddings to construct a demand-conditioned semantic space.
	LOC-ZSON [31]	2024	Builds object-centric semantic representations and uses LLM prompting to achieve zero-shot language–vision alignment in open-vocabulary settings.
	CrossText2Loc [32]	2025	Enhances cross-view geo-semantic alignment by extending CLIP with positional encoding, OCR grounding, and hallucination suppression mechanisms.
	VLFly [33]	2025	Integrates LLaMA-3 semantic encoding with CLIP-based visual grounding to support open-vocabulary goal understanding.
Instruction Semantic Parsing	InstructNav [34]	2024	Parses natural language instructions into Dynamic Chains of Navigation, providing a structured and executable semantic representation for navigation.
	Long et al. [35]	2024	Refines instruction semantics through multi-expert LLM discussions, producing more reliable and consistent semantic interpretations before navigation.
	NaviLLM [28]	2024	Unifies navigation, localization, summarization, and QA within a schema-based generative semantic parsing framework.
	GC-VLN [36]	2025	Parses natural language into entity–spatial graph constraints, enabling training-free semantic-to-action mapping through structured instruction graphs.
	Wang et al. [37]	2025	Extracts instruction-related semantic cues and aligns them with candidate paths on 3D semantic maps, enabling instruction-aware path planning.

**Table 2:** Representative methods for environment understanding in VLN.

Category	Methods	Year	Contributions
Feature Extraction	LangNav [38]	2023	Integrates VLM and detectors to form language-aligned semantic perception for complex environments.
	Dorbala et al. [39]	2023	Uses LLM commonsense priors to infer object locations and guide zero-shot exploration.
	ESC [40]	2023	Applies soft commonsense constraints to improve semantic-driven search in unseen scenes.
	MapGPT [27]	2024	Transforms visual observations into map-guided prompts to support LLM-based planning.
	Qiu et al. [41]	2024	Builds open-vocabulary 3D semantic maps unifying spatial and language-driven navigation cues.
	LLVM-Drone [42]	2025	Extracts semantic environmental features through LLM–vision collaboration with structured prompts and consistency checks.
	LM-Nav [43]	2023	Aligns CLIP visual features with textual landmark descriptions for accurate goal grounding.
	AeroDuo [45]	2025	Combines high-altitude semantic mapping with low-altitude obstacle-aware perception for dual-view UAV scene understanding.
	NaviD [46]	2024	Uses EVA-CLIP temporal encoding to produce continuous scene representations from video history.
	NavGPT-2 [47]	2024	Projects visual frames into lightweight fixed-length tokens for efficient reasoning.
	NavAgent [48]	2024	Fuses multi-scale urban street-view cues using GLIP-enhanced landmark extraction and cross-view matching to improve semantic grounding.
	FLAME [49]	2025	Learns multi-view semantic representations via Flamingo-based multimodal fusion, supporting route summarization and end-to-end action prediction.
	EZREAL [50]	2025	Improves far-distance target localization using multiscale visual cues, visibility-aware memory, and depth-free heading estimation.
	Li et al. [51]	2023	Predicts future-view semantic cues to enhance forward-looking visual understanding.
Structured Memory	OVER-NAV [52]	2024	Constructs an Omnigraph using LLM-parsed semantics and open-vocabulary detections as a persistent structured memory for IVLN.
	CityNavAgent [53]	2025	Uses hierarchical semantic planning with global memory to support long-range UAV navigation.
	JanusVLN [54]	2025	Separates semantic and spatial cues via dual implicit memories to improve generalization.
	COSMO [55]	2025	Uses selective sparse memory to reduce redundancy and enhance cross-modal efficiency.
	Guide-LLM [56]	2024	Represents spatial topology as text-based nodes/edges for LLM-accessible world memory.
	Dynam3D [57]	2025	Introduces dynamic layered 3D tokens enabling semantic–geometric coupling and long-term reusable 3D environment memory.
	VLN-KHVR [58]	2025	Integrates external knowledge and navigation history into a temporally coherent state memory.
	VLN-ChEnv [59]	2025	Models evolving environments via temporal multi-modal updates for dynamic scene understanding.
	StreamVLN [60]	2025	Uses SlowFast streaming memory to capture short-term changes while retaining long-term context.
Generative Prediction	VLFM [61]	2024	Builds vision–language frontier maps to predict semantic frontiers in unseen environments.
	VISTA [62]	2025	Uses diffusion-based imagination to generate future scene hypotheses for proactive navigation.
	Fan et al. [63]	2025	Uses scene-map prompts to generate task-aligned navigation instructions.
	ImagineNav [64]	2024	Prompts VLMs to imagine and align future states to guide anticipatory navigation decisions.
	Saanum et al. [65]	2024	Demonstrates simple models can reliably predict future states.
	KiteRunner [66]	2025	Combines language parsing, UAV mapping, and diffusion-based local trajectory generation for outdoor navigation.

**Table 3:** Representative methods for high-level planning in VLN.

Category	Methods	Year	Contributions
Explicit Reasoning	EvolveNav [67]	2025	Improves navigation via self-evolving LLM reasoning, repeatedly refining explicit reasoning chains to enhance embodied decision-making.
	MSNav [68]	2025	Performs explicit LLM-based spatial reasoning with dynamic memory to support zero-shot navigation in unseen environments.
	NavGPT [26]	2024	Performs explicit language-driven reasoning by integrating textualized observations with structured step-by-step decision chains.
	NavCoT [69]	2025	Introduces navigational CoT via imagined next-view prediction and disentangled reasoning for improved action selection.
	SayCan [70]	2022	Combines LLM-generated high-level subgoals with affordance-based feasibility grounding for reliable execution.
	Khan et al. [71]	2025	Uses context-aware LLM reasoning with weighted goal fusion and interpretable direction scoring for robust UAV navigation.
	VLN-Zero [72]	2025	Employs neuro-symbolic scene-graph planning with rapid exploration and cache-enabled reasoning for zero-shot transfer.
	FSR-VLN [73]	2025	Uses hierarchical multi-modal scene graphs with fast-to-slow reasoning for interpretable and efficient navigation planning.
	Open-Nav [74]	2025	Leverages open-source LLMs for waypoint prediction and spatiotemporal CoT reasoning in continuous VLN.
Implicit Reasoning	NavGPT-2 [47]	2024	Uses lightweight visual tokenization for efficient latent-space VLN reasoning.
	Kang et al. [75]	2025	Reduces inference redundancy through dynamic early-exit and adaptive reasoning depth without explicit planning chains.
	Liu et al. [76]	2024	Learns implicit state-action distributions via energy-based modeling for stable, distribution-aligned navigation policies.
	VLN-R1 [77]	2025	Applies reinforcement fine-tuning on LVLM agents to optimize long-horizon behavior through reward-driven policy refinement.
	PGNS [78]	2024	Learns pixel-guided navigation skills that implicitly couple visual cues with action generation for zero-shot object navigation.
	AerialVLN [79]	2023	Learns continuous UAV navigation policies through cross-modal fusion without explicit reasoning chains.
Generative Planning	Cog-GA [80]	2024	Builds a generative LLM-based agent with cognitive maps enabling semantic memory, reflective correction, and long-horizon planning.
	Ha et al. [81]	2018	Establishes latent-dynamics imagination frameworks inspiring generative planning for later VLN world-model approaches.
	Dreamwalker [82]	2023	Constructs a discrete abstract world model enabling mental planning and multi-step rollout in continuous VLN environments.
	DreamNav [83]	2025	Utilizes trajectory-based imagination with view correction and future-trajectory prediction for zero-shot long-range planning.
	Bar et al. [84]	2025	Uses controllable video-generation world models to simulate future observations for planning in familiar and novel environments.

**Table 4:** Representative methods for low-level control in VLN.

Category	Methods	Year	Contributions
Basic Control Strategies	Kåsene et al. [85]	2025	Compares egocentric low-level actions against panoramic actions, showing finer semantic-behavior alignment.
	NaviD [46]	2024	Video-based VLM performs end-to-end continuous control for unified language–vision–action next-step planning.
	SayNav [86]	2024	Converts each LLM-generated planning step into short-range point-goal tasks, enabling execution through basic low-level control commands.
	Chen et al. [87]	2025	Uses semantic segmentation and affordance-grounding to map linguistic intent into precise continuous control actions.
	UAV-ON [88]	2025	Defines an open-world UAV object-goal benchmark that evaluates basic action-level control policies.
	RT-2 [89]	2023	Unifies vision–language representations to generate executable action tokens via large-scale robotic transformer training.
	OpenVLA [90]	2024	Open-source vision–language–action framework producing end-to-end action tokens through shared multimodal embeddings.
	LaViRA [91]	2025	Translates natural-language instructions to robot-level continuous actions via unified language–vision–action generation.
	Lagemann et al. [92]	2023	Learns invariant latent dynamics enabling multi-step internal state prediction for improved control consistency.
Closed-loop Control	SkyVLN [93]	2025	Integrates VLN perception with NMPC to perform closed-loop continuous control for UAV navigation.
	UAV-VLN [94]	2025	Employs multimodal feedback loops enabling real-time trajectory correction for UAV-based VLN in complex outdoor scenes.
	Narrate2Nav [95]	2025	Embeds implicit language reasoning into visual encoders, enabling human-aware real-time control in dynamic environments.
	CL-CoTNav [96]	2025	Combines hierarchical chain-of-thought with confidence-triggered re-reasoning to achieve self-correcting closed-loop actions.
	LogisticsVLN [97]	2025	Lightweight multimodal-LLM cascade loop that on-the-fly corrects window-level positioning errors in UAV terminal delivery.
	Choutri et al. [98]	2025	Offline bilingual voice feedback loop enabling zero-latency semantic-control self-correction in low-connectivity dynamic outdoors.
	MMCNav [99]	2025	Multi-agent dual-loop reflection closed-loop that cooperatively refines long-range dense-instruction outdoor trajectories in real time.
Generative Control	Shi et al. [100]	2025	Combines diffusion policies with DAgger to reduce compounding errors and stabilize long-horizon action generation.
	NavDP [101]	2025	Generates multiple candidate trajectories in latent space and refines them via privilege-informed critic-guided optimization.
	ComposableNav [102]	2025	Uses composable diffusion models to generate flexible, instruction-aligned continuous control sequences in dynamic settings.
	FLUC [103]	2025	LLM offline generates flight-control code, turning natural language into executable aerial logic for zero-manual-programming generative UAV control.

**Table 5:** Summary of efficient and edge-deployable VLN/LLM-based navigation systems.

Methods	Base Model	Deployment Platform	Inference Latency	Task Performance
TinyVLA [174]	Pythia-0.4–1.3 B	A6000 (training only)	5 ms/step	↑ +25.7% SR
EdgeVLA [175]	Qwen2-0.5 B + SigLIP + DINO	A100 (training only)	14 ms/action	≈OpenVLA, 7× faster
Lite VLA [176]	SmolVLM-256 M	Raspberry Pi 4 (4 GB)	0.09 Hz	Stable office nav
Gurunathan et al. [177]	LLaVA-1.5–7 B	Jetson Orin NX/Nano	19.25 tok/s	>90% VQA acc
GRaD-Nav++ [178]	BLIP-2 6.7 B + 3D-Gaussian + Diff-dynamics	Jetson Orin NX 16 GB	45 ms/step	↑ +18.6% SR (Urban-VLN)
EfficientNav [179]	LLaMA-3.2–11 B/LLaVA-34 B	Jetson AGX Orin (32 GB)	0.35 s/step	↑ +11.1% SR (Habitat)
SINGER [180]	CLIP-ViT + SV-Net	Jetson Orin Nano (8 GB)	12 Hz infer	↑ +23.3% SR
PanoGen++ [181]	Stable Diffusion	Offline generation	×	↑ +1.77% SR (R2R)
PEAP-LLM [182]	Llama-2–7 B	RTX 3090 (training only)	823 ms/step	↑ +4.0% SPL (REVERIE)
VLN-PETL [183]	BERT+ViT	GPU training only	×	≈Full fine-tune
VL-Nav [184]	CLIP-Res50 + Spatial-LLM-7 B	Jetson Xavier NX	55 ms/frame	+12.3% SR (Habitat-R2R)
ClipRover [185]	CLIP ViT-B/32	Raspberry Pi 4	0.11 s/obs	86% zero-shot target discovery

Note. SR (Success Rate), SPL (Success weighted by Path Length), and other task-specific KPIs used in this table will be formally defined in the evaluation section. ↑ indicates improved performance, and × indicates that the corresponding information was not explicitly reported.

**Table 6:** Snapshot summary of task datasets for VLN.

Dataset	Snapshot Description
R2R 2018 [4]	Content: 90 different building scenes from the Matterport3D dataset (including homes, offices, churches, etc.), with 21 k detailed natural language navigation instructions. Highlights: the first dataset that connects natural language instructions with large-scale, real 3D environments, driving a paradigm shift in VLN from grid worlds to real-world scenarios. Limitations: The path is relatively simple, lacking interactive tasks and dynamic environments.
RoomNav 2018 [186]	Content: 45 K+ indoor 3D environments in House3D with room categories, agent viewpoints, and navigation trajectories paired with concise, room-type textual instructions. Highlights: It offers structured, goal-directed navigation tasks that tightly couple semantic room labels with embodied visual exploration. Limitations: Instructions and goals are simplistic and low-level, limiting linguistic richness and real-world navigation generalization.
R4R 2019 [187]	Content: 200 K instructions in 61 scenes (train) plus 1000+ (val-seen) and 45,162 (val-unseen) split. Highlights: It concatenates adjacent R2R trajectories to form longer, twistier paths, reducing bias toward shortest-path behavior. Limitations: Because paths are algorithmically combined rather than naturally annotated, some linguistic fidelity may still be imperfect or unnatural.
RxR 2020 [188]	Content: 120 K+ multilingual (English, Hindi, Telugu) navigation instructions and 16,000 distinct paths in Matterport3D scenes. Highlights: It provides dense spatiotemporal grounding by aligning each word in the instruction with the speaker’s pose trajectory, and supports multilingual VLN. Limitations: High complexity, high resource demands, occasional instruction–trajectory misalignment.
CVDN 2020 [189]	Content: 2 K human–human dialogs spanning over 7000 navigation trajectories across 83 Matterport houses. Highlights: It enables interactive navigation by incorporating dialog-based grounded guidance where a navigator asks questions and an oracle gives privileged-step advice. Limitations: Dialog history can be noisy and sparse, making it challenging for agents to infer correct actions purely from conversational context.
VLN-CE 2020 [190]	Content: 16 K+ path-instruction pairs across 90 Matterport3D scenes. Highlights: Continuous-motion navigation in realistic 3D environments rather than discrete graph steps. Limitations: Requires fine-grained control and is more computationally demanding, making training and sim-to-real transfer harder.
REVERIE 2020 [5]	Content: 21,000 human-written high-level navigation instructions across 86 buildings, targeting 4 K remote objects. Highlights: It combines navigation with object grounding, requiring an agent not only to walk but also to identify a distant target object. Limitations: The high-level, concise instructions make precise step-by-step navigation hard, and locating the correct object in complex scenes is challenging.
ALFRED 2020 [191]	Content: 25 K+ English directives paired with expert demonstrations across 120 indoor AI2-THOR scenes. Highlights: It supports long-horizon, compositional household tasks with both high-level goals and step-by-step instructions, combining navigation and object manipulation. Limitations: The tasks are very complex and long, making models hard to train and generalize, and success rates remain low.
SOON 2021 [192]	Content: 3000 natural-language instructions and 40 K trajectories across 90 Matterport3D scenes. Highlights: It emphasizes starting-point independence and coarse-to-fine scene descriptions, so an agent can navigate from anywhere to a fully described target. Limitations: Dense, complex instructions and long trajectories make navigation hard to follow.
HM3D 2021 [193]	Content: Over 1000 high-quality, photorealistic indoor 3D reconstructed environments covering diverse residential and commercial buildings. Highlights: The largest and most realistic indoor 3D reconstruction dataset used in embodied AI; provides dense geometry, consistent semantics, and significantly richer diversity than previous scanned datasets. Limitations: Contains only environment scans—no human-written navigation instructions; must be paired with VLN task datasets for instruction grounding.
HM3D-SEM 2023 [194]	Content: Semantic extension of HM3D, including instance-level annotations, room categories, object labels, and spatial relationships. Highlights: Supports semantic-driven VLN, object-centric navigation, and open-vocabulary reasoning by providing detailed scene semantics. Limitations: Like HM3D, it lacks natural-language instructions and requires external datasets for grounded VLN tasks.
DDN 2023 [6]	Content: 1000+ demand-instructions mapped to 600 AI2-THOR + ProcThor scenes. Highlights: It lets agents reason over user needs (e.g., “I’m thirsty”) instead of object names, finding any object whose attributes satisfy the demand. Limitations: Fixed mappings limit generalization to unseen environments.
Talk2Nav 2021 [195]	Content: 10 K human-written navigation routes over 40 K Google Street View nodes in a 10 km × 10 km area of New York City. Highlights: It captures long-range, real-world outdoor navigation grounded in verbal instructions referencing landmarks and directions. Limitations: Ambiguous scenes and instructions make it hard to reliably localize and follow instructions.

**Table 7:** (**a**). Summary of representative indoor simulation environments. (**b**). Unified comparison criteria for simulator selection.

(**a**)				
**Simulators**	**Support Scene**		**Highlights**	
Matterport3D [196]	Realistic, photo-quality indoor environments like homes, offices, and public spaces		Interactive 3D navigation with accurate visual and spatial cues; Supports advanced embodied AI research	
House3D [186]	Diverse realistic indoor environments like multi-room houses and apartments for navigation tasks.		Supports realistic 3D navigation with visual grounding, enabling agents to understand and interact with complex indoor spaces	
Habitat [198]	Diverse large-scale indoor environments like apartments, offices, and malls for immersive navigation tasks.		High-quality, photorealistic 3D environments with realistic physics; supports complex visual navigation and interaction with objects	
AI2-THOR [199]	Diverse rendered/synthetic hand-modeled indoor scenes		Object manipulation and agent interaction; supports tasks that combine navigation with physical actions	
RoboTHOR [200]	Realistic indoor apartment-style scenes paired with physical counterparts		Provides matched simulated and real environments for evaluating Sim2Real transfer; supports embodied navigation and object-search tasks	
StreetNav [201]	Real-world urban street-view imagery from cities such as New York and London		Enables real-time navigation with sequential street-view observations; designed for studying language-guided urban navigation and human–agent interaction	
AirSim [202]	Photorealistic outdoor and urban environments with UAV and ground-vehicle support		Offers high-fidelity visual and physical simulation using Unreal Engine; supports continuous-control aerial navigation, obstacle avoidance, and autonomous driving research	
(**b**)				
**Platform**	**Domain**	**Scene source**	**Interaction/Physics**	**Sim2Real relevance**
Matterport3D [196]	Indoor	Real reconstruction	✘	✘
House3D [186]	Indoor	Synthetic	✘	✘
Habitat [198]	Indoor	Platform (supports scanned scenes)	✔	△
AI2-THOR [199]	Indoor	Synthetic hand-modeled	✔	✘
RoboTHOR [200]	Indoor	Synthetic + paired real	✔	✔
StreetNav [201]	Urban	Street-view imagery	✘	✘
AirSim [202]	Outdoor/Aerial	Unreal Engine	✔	△

“✔ indicates explicit support; △ indicates transfer-friendly but without paired real-world counterparts; and ✘ indicates no explicit support.”.

**Table 8:** Main path-level indicators in VLN evaluation.

Indicators	Description
SR [203]	The percentage of intelligent agents that successfully reach their goals within the tolerance range
TL [204]	The average path length of the agent is used to evaluate path efficiency
SPL [203]	The most commonly used comprehensive indicator, taking into account both success rate and path optimality
OSR [4]	Measures the percentage of agents that enter the target area at least once, regardless of path efficiency
NE [4]	The shortest-path distance between the agent’s final stopping point and the target position

**Table 9:** Main Semantic-level indicators in VLN evaluation.

Indicators	Description
NDTW [205]	Measure the temporal and spatial matching degree between the predicted trajectory and the reference trajectory
SDTW [205]	Measure the temporal and spatial matching degree between the predicted trajectory and the reference trajectory
CLS [187]	Assess the path–language consistency jointly via coverage and length constraints
GP	Measure the average percentage of progress an agent makes before reaching its goal

## Data Availability

All data are available upon request.
